# Identification of miR-10b, miR-26a, miR-146a and miR-153 as potential triple-negative breast cancer biomarkers

**DOI:** 10.1007/s13402-015-0239-3

**Published:** 2015-09-21

**Authors:** Insaf Fkih M’hamed, Maud Privat, Flora Ponelle, Frédérique Penault-Llorca, Abderraouf Kenani, Yves-Jean Bignon

**Affiliations:** Département d’Oncogénétique, Centre Jean Perrin, BP 392, 63011 Clermont-Ferrand, France; EA4677 ERTICA, Université d’Auvergne, Clermont-Ferrand, France; Faculté de médecine de Monastir, Laboratoire de Biochimie, Unité de recherche UR 12ES08 “Signalisation Cellulaire et Pathologies”, 5019 Monastir, Tunisie

**Keywords:** MicroRNAs, Human triple-negative breast cancer cells, BRCA1, Regulation

## Abstract

**Background:**

Familial triple-negative breast cancers are often linked to mutations in the *BRCA1* tumor suppressor gene. In sporadic triple-negative breast cancers *BRCA1* is frequently inactivated at the transcriptional level, and it has been reported that this inactivation may be brought about by promoter methylation. More recently, it was found that *BRCA1* may also be regulated at the post-transcriptional level by miRNAs. Here, we explored the expression of putative *BRCA1*-regulating miRNAs in sporadic human triple-negative breast cancer cells.

**Methods:**

Nine sporadic human breast cancer-derived cell lines and one benign breast epithelium-derived cell line were assessed for their hormone receptor, growth factor receptor and cytokeratin status by immunocytochemistry. The expression of 5 selected miRNAs predicted to target *BRCA1* was assessed using qRT-PCR in the 10 cell lines. In addition, expression profiles of 84 known breast cancer-associated miRNAs were established in these 10 cell lines using PCR Array and qRT-PCR, respectively. The putative role of pre-selected candidate miRNAs in breast cancer development was assessed through exogenous expression of these miRNAs and their anti-miRNAs (‘antagomirs’) in MDA-MB-231 and MCF7 breast cancer-derived cells.

**Results:**

Based on our expression profiling results, four candidate miRNAs (miR-10b, miR-26a, miR-146a and miR-153) were selected as being potentially involved in triple-negative breast cancer development. Exogenous expression assays revealed that miR-10b and miR-26a, but not miR-146a, can down-regulate the expression of *BRCA1* in both triple-negative MDA-MB-231 and luminal epithelial MCF7 breast cancer-derived cells, whereas miR-153 could down-regulate *BRCA1* expression only in MCF7 cells. *In silico* analysis of The Cancer Genome Atlas (TCGA) data confirmed that miR-146a is significantly higher expressed in triple-negative breast tumors compared to other (non triple-negative) breast tumors.

**Conclusion:**

Our work provides evidence for the involvement of specific miRNAs in triple-negative breast cancer development through regulating *BRCA1* expression.

**Electronic supplementary material:**

The online version of this article (doi:10.1007/s13402-015-0239-3) contains supplementary material, which is available to authorized users.

## Introduction

Breast cancer is the most commonly diagnosed cancer in women (23 % of all cancers), with an estimated diagnosis of 1.38 million new cases each year worldwide [1–4]. At the molecular level, breast cancers can be classified in four main subtypes, depending on the expression of hormone and growth factor receptors. The luminal subtypes A and B express both the estrogen receptor (ER) and the progesteron receptor (PR), the HER2-enriched subtype shows amplification and over-expression of the human epidermal growth factor receptor 2 (HER2), and the triple-negative subtype does not express any of these receptors. This classification correlates well with the prognosis, ranging from good for luminal A tumors to poor for the triple-negative tumors [5, 6]. The latter category, representing ~10 to 15 % of all breast cancers, is generally considered aggressive [7, 8].

Triple-negative breast cancers are often linked to inactivation of the *BRCA1* tumor suppressor gene. In familial breast cancer, 85 % of the *BRCA1* mutated tumors are triple-negative [9, 10]. In sporadic triple-negative breast cancers, *BRCA1* is frequently inactivated at the transcriptional level, and it has been shown that *BRCA1* inactivation may be due to methylation of its promoter brought about by ID4 [11, 12]. More recently it has been found that *BRCA1* may also be regulated at the post-transcriptional level by microRNAs [13].

MicroRNAs (miRNAs) are small non-coding 19–25 nucleotide-long RNAs that can post-transcriptionally regulate gene expression by binding to the 3’untranslated regions (3′-UTR) of target messenger RNAs (mRNAs), thereby leading to mRNA degradation or translational repression [14–16]. miRNAs have been shown to be involved in diverse biological processes [17–19]. In human breast cancer it has been shown that they can act either as tumor suppressors (i.e., miR-206, miR-17-5p, miR-125a, miR-125b, miR-200, let-7, miR-34 and miR-31) or as oncogenes (i.e., miR-21, miR-155, miR-10b, miR-373 and miR-520c) [20]. Previously, it has been shown that *BRCA1* gene expression is relatively low in ER-negative and high-grade breast cancers [21] and that *BRCA1* gene expression is significantly down-regulated in triple-negative breast cancers [22]. The number of miRNAs that may regulate the expression of *BRCA1* or that may serve as transcriptional targets of *BRCA1* is rapidly increasing. Considering the tumor-suppressive role of *BRCA1*, any perturbation in this regulatory function is likely to have an effect on *BRCA1*-mediated tumor development [23]. Moskwa et al. reported that miR-182-mediated down-regulation of *BRCA1* can impede DNA repair and, as such, affect breast cancer therapy [24]. It has also been shown that miR-146a and miR-146b-5p can down-regulate the expression of the *BRCA1* gene in triple-negative sporadic breast cancers [18]. In contrast, a recent study reported that miR-146a expression levels increased simultaneously with *BRCA1* expression levels. In addition, it was suggested that post-transcriptional regulation of epidermal growth factor receptor (*EGFR*) expression by *BRCA1* may be mediated by miR-146a [25].

Here, we investigated miRNA expression profiles in nine human breast cancer-derived cell lines and one benign breast epithelium-derived cell line. By doing so, we identified a set of miRNAs putatively involved in *BRCA1* gene expression regulation and breast cancer development.

## Materials and methods

### Cell culture

Nine selected human breast cancer-derived cell lines and one benign breast epithelium-derived cell line (Table 1) were cultured in a humified incubator at 37 °C containing 5 % CO2. Cell line MCF10a was obtained from the American Type Culture Collection (ATCC; Manassas, VA, USA) and was cultured in DMEM/F12 medium (Invitrogen Life Technologies, Carlsbad, CA, USA) supplemented with 10 % horse serum, 20 ng/ml EGF, 100 ng/ml cholera toxin, 500 ng/ml hydrocortisone, 2 mM L-glutamine and 20 ng/ml gentamicin.

**Table 1:** Human breast cancer cell lines and their characteristics

				Immunocytochemistry						
Cell lines			BRCA1 status	ER	PR	HER2	ck5/6	CK14	EGFR	cKIT
MCF10A	Non tumoral	Benign	wild-type	–	–	–	+ (90 %)	+ (80 %)	+ (100 %)	–
MCF7	Tumoral	Luminal	wild-type	90 % (1+)	40 % (1/2+)	–	–	–	–	–
T47D	Tumoral	Luminal	wild-type	50 % (1/2+)	80 % (1/2/+)	–	–	–	+(40 %)	–
MDA-MB-231	Tumoral	TN	wild-type	–	–	–	–	–	+ (100 %)	–
HCC1937	Tumoral	TN	5382insC	–	–	–	+ (25 %)	+ (20 %)	+ (100 %)	–
MDA-MB-436	Tumoral	TN	5396 + 1G > A	–	–	–	–	–	+ (90 %)	–
SUM149PT	Tumoral	TN	2288delT	–	–	–	+ (25 %)	+ (<1 %)	+ (100 %)	–
SUM1315MO2	Tumoral	TN	185delAG	–	–	–	–	–	+ (90 %)	–
SUM1315-LXSN	Tumoral	TN	185delAG	–	–	–	–	–	+ (60 %)	–
SUM1315-BRCA1	Tumoral	TN	185delAG + WT	–	–	–	–	–	+ (80 %)	–

TN: Triple-Negative. The BRCA1mutational status as also the Estrogen Receptor (ER), Progesterone Receptor (PR), HER2 protein, Cytokeratin5/6 (ck5/6), Cytokeratin14 (ck14), Epidermal Growth Factor Receptor (EGFR) and cKIT protein expression status are indicated

Cell lines MCF7, T47D, MDA-MB-231, HCC1937 and MDA-MB-436 were also obtained from the ATCC and were cultured in RPMI-1640 medium (Invitrogen Life Technologies, Carlsbad, CA, USA) supplemented with 10 % fetal bovine serum (FBS), 2 mM L-glutamine and 20 ng/ml gentamicin. Cell lines SUM149PT and SUM1315MO2 were obtained from Asterand (Royston, Hertfordshire, UK) and were grown in Ham’s F12 medium according to the supplier’s instructions. Cell lines SUM1315-LXSN and SUM1315-BRCA1 were derived from the *BRCA1* mutated (185delAG) SUM1315MO2 cell line, stably transfected with empty LXSN or LXSN-BRCA1 plasmids, respectively. The *BRCA1* mutation status of the other cell lines has previously been reported by Elstrodt et al. [26].

### Immunocytochemistry

Cells were fixed in Preservcyt solution (Thinprep) and cytoblocks were prepared using a Shandon Cytoblock kit (Thermo Scientific). The estrogen receptor (ER), progesterone receptor (PR), human epidermal growth factor receptor 2 (HER2), cytokeratin5/6, cytokeratin14, epidermal growth factor receptor (EGFR) and tyrosine kinase receptor cKIT protein status was determined by immunocytochemistry on 3 μm-thick sections. Immunostainings were performed in a BenchmarkXT fully automatized stainer (Ventana Medical Systems) and scored semi-quantitatively by an expert pathologist under a light microscope.

### MiRNA target prediction algorithms

Five available algorithms predicting miRNA-mRNA interactions were used to select miRNAs targeting the 3′-UTR of the *BRCA1* gene transcript: TargescanHuman (http://www.targetscan.org/vert_61/), Tarbase (http://diana.imis.athenainnovation.gr/DianaTools/index.php?r=tarbase/index), Miranda (http://www.microrna.org/microrna/home.do), MicroCosmTargets (http://www.ebi.ac.uk/enright-srv/microcosm/htdocs/targets/v5/) and.PicTar (http://pictar.mdc-berlin.de/cgi-bin/PicTar_vertebrate.cgi).

For all algorithms we used the default parameters and we selected miRNAs that putatively target *BRCA1* by at least three algorithms.

### RNA extraction, reverse transcription and quantitative real-time PCR

Total cell RNA was extracted using QIAzol reagent (Qiagen miRNeasy mini kit) according to the manufacturer’s instructions. The RNA quality and yields were assessed using an Agilent Bioanalyzer 2100 (Kit Agilent RNA 6000 nano) in conjunction with a spectrophotometer. One microgram of total RNA was reverse-transcribed using a miscript II RT kit (Qiagen, France). 15 nanograms of cDNA was used in triplicate for quantitative real-time PCR using a miscript SYBER Green PCR kit (Qiagen, France) on an ABI7900HT system and an Applied Biosystems ViiA™ 7 Real-Time PCR system. Selected miRNA primers were obtained from miScript Primer Assay (Qiagen, France) and are listed in supplementary Table S1. U6 (Hs-RNU6-2–1 miscript Primer Assay) and 18S rRNA were used as internal controls. All samples were normalized to the internal controls and fold changes were calculated through relative quantification (RQ = 2^-∆∆CT^).

### PCR array-based profiling

The Human Breast Cancer miScript miRNA PCR Array (MTHS-109ZE-4) was used according to the manufacturer’s protocol (Qiagen, France) for the profiling of 84 miRNAs known or predicted to alter in expression during breast cancer initiation and/or progression. The protocol enables real-time PCR profiling of mature miRNA on an ABI7900HT system in combination with a miScript SYBR Green PCR kit, which contains a miScript Universal Primer (reverse primer), and a QuantiTect SYBR Green PCR Master Mix. 250 nanogram of total RNA was reverse-transcribed using a miscript II RT kit (Qiagen).

### Transfection assays

Selected miRNA mimics and miRNA inhibitors (‘antagomirs’) were obtained from miScript miRNA Mimic and miScript miRNA Inhibitor, respectively (Qiagen, France) and are listed in supplementary Table S2. The siRNA used for inhibiting *BRCA1* gene expression (siBRCA1) was synthesized by Thermo Scientific, Dharmacon (M-003,461–02–0005, siGENOME SMART pool, Human BRCA1 (672), 5 nmol). Cells were seeded at a density of 300,000 cells per well in 6-well plates. Twenty-four hours later, miRNAs and siRNAs were transfected using Lipofectamine 2000 reagent (Invitrogen, CA, USA) following the manufacturer’s instructions. As a mock control, cells were transfected with transfection reagent alone (Tmock). Three days after transfection, the cells were washed with PBS and recovered for further analysis.

### Cell proliferation assay

MDA-MB-231 cells and MCF7 cells were collected, seeded at a density of 3000 cells per well into a 96-well plate and cultured in a humified incubator at 37 °C containing 5 % CO2 for 24 h. Subsequently, the cells were transfected with Tmock (transfection reagent alone), siBRCA1, miR-146a, anti-miR-146, miR-153, miR-10b and miR-26a using Lipofectamine 2000 reagent (Invitrogen, CA, USA) according to the manufacturer’s protocol. After 48 h *in vitro* cell proliferation was evaluated using CCK-8 (Cell Counting Kit-8, Sigma-Aldrich) according to the manufacturer’s instructions. The absorbance was determined at 450 nm using a microplate reader. All experiments were performed in triplicate.

### *In silico* analysis of miRNAs using TCGA

Both clinical and miRNA sequencing data of invasive breast cancers were downloaded from The Cancer Genome Atlas (TCGA) database. A total of 519 patients with information on ER, PR and HER2 status were selected to compare the expression profiles of four selected miRNAs (miR-10b, miR-26a, miR-146a and miR-153) in the respective tumors. 88 cases were found to have a negative ER, PR and HER2 (i.e., triple-negative) phenotype, whereas 431 cases were positive for at least one of these receptors.

### Statistical analysis

Student’s *t-*test was used to assess statistical differences in mean expression between groups. A *p*-value ≤ 0.05 was considered statistically significant.

## Results

### miR-10b and miR-26a are preferentially expressed in triple-negative breast cancer-derived cell lines

**Fig. 1:**
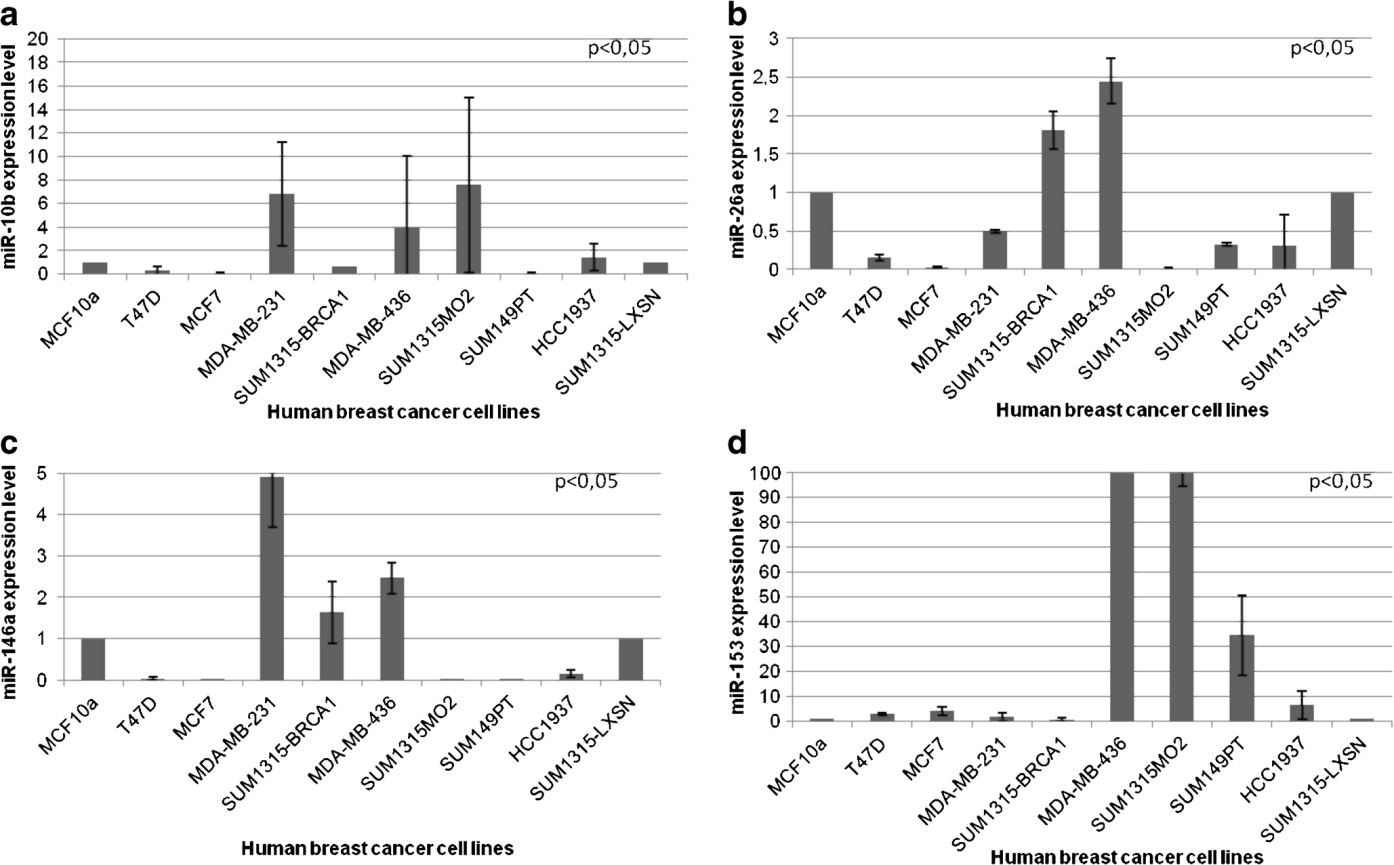
Expression of miRNAs in mammary cell lines. (a) miR-10b expression level, (b) miR-26a expression level, (c) miR-146a expression level and (d) miR-153 expression level. miRNA expression was determined by qRT-PCR in one benign mammary epithelium cell line (MCF10a) and nine tumor cell lines (two luminal cell lines: MCF7 and T47D and seven triple-negative cell lines: MDA-MB-231, SUM1315MO2, SUM1315-LXSN, SUM1315-BRCA1, MDA-MB-436, SUM149PT and HCC1937). Expression of miRNA was normalized using U6. A *p*-value < 0.05 is considered significant (between triple-negative and luminal cell lines)

In order to identify miRNAs implicated in triple-negative breast cancer, we used a miScript miRNA PCR Array to profile the expression of 84 miRNAs considered to be involved in breast tumorigenesis. Ten cell lines were analyzed: one benign breast epithelium-derived cell line (MCF10a), two luminal breast cancer-derived cell lines (MCF7, T47D) and seven triple-negative breast cancer-derived cell lines (MDA-MB-231, MDA-MB-436, HCC1937, SUM149PT, SUM1315MO2, SUM1315-BRCA1 and SUM1315-LXSN). The cell lines were characterized by immunocytochemistry as presented in Table 1. In order to reveal whether particular miRNAs are specifically involved in triple-negative breast cancers, we compared their expression in the respective cell line subgroups. For this analysis we focused on six miRNAs that were found to be differently expressed in at least two subgroups by PCR Array, i.e., miR-10b, miR-15b, miR-26a, miR-155, miR-206 and miR-485-5p (Table 2, Table S3). To validate the PCR Array results, we performed qRT-PCR analysis of the six selected miRNAs in the 10 cell lines. Only two of these miRNAs (i.e., miR-10b and miR-26a) were found to be well-expressed in these cell lines. The other miRNAs were expressed at low levels or were not detectable at all. miR-10b was highly expressed in 3 triple-negative breast cancer cell lines (MDA-MB-231, MDA-MB-436 and SUM1315MO2) (Fig. 1a). miR-26a was found to be expressed in all 10 cell lines, and to be higher expressed in the triple-negative cell lines than the luminal cell lines (MCF7, T47D) (Fig. 1b).

**Table 2:** Profiling of miRNAs in human breast cancer cell lines by miScript miRNA PCR Array. Subgroups of cell lines are compared

miRNA (*p* < 0.05)	SUM1315-LXSN / SUM1315-BRCA1	Basal BRCA1+/ Luminal	Basal BRCA1- / Luminal	Tumoral / Benign	Basal BRCA1- / Benign	Basal BRCA1+ / Benign
Over-Expressed (Fold change >2)	**hsa-miR-206**	**hsa-miR-10b**	**hsa-miR-26a**	**hsa-miR-485-5p**	**hsa-miR-485-5p**	hsa-miR-100
	**hsa-miR-10b**	hsa-miR-100				hsa-miR-148a
	hsa-miR-129-5p	**hsa-miR-26a**				hsa-miR-328
						hsa-miR-607
Under-Expressed (Fold change <0.5)	hsa-miR-16	hsa-let-7e	**hsa-miR-15b**	hsa-let-7b	hsa-let-7f	hsa-miR-125b
	hsa-miR-31	hsa-miR-19a	hsa-miR-328	hsa-let-7f	hsa-miR-140-5p	hsa-miR-128
	hsa-miR-203	**hsa-miR-206**	**hsa-miR-485-5p**	hsa-miR-140-5p	**hsa-miR-155**	**hsa-miR-155**
	hsa-miR-328	hsa-miR-429	hsa-miR-489	**hsa-miR-155**	**hsa-miR-15b**	hsa-miR-193b
	hsa-miR-607	**hsa-miR-485-5p**	hsa-miR-548c-3p	hsa-miR-193b	hsa-miR-210	hsa-miR-195
	hsa-miR-613	hsa-miR-497		**hsa-miR-26a**	hsa-miR-25	hsa-miR-199b-3p
				hsa-miR-26b	**hsa-miR-26a**	hsa-miR-199a-5p
				hsa-miR-29c	hsa-miR-26b	hsa-miR-19a
					hsa-miR-27a	hsa-miR-205
					hsa-miR-29c	**hsa-miR-206**
					hsa-miR-489	hsa-miR-20a
					hsa-miR-495	hsa-miR-214
						hsa-miR-25
						**hsa-miR-26a**
						hsa-miR-26b
						hsa-miR-27a
						hsa-miR-29a

Bold entries are miRNAs that were found to be differently expressed in at least two subgroups by PCR Array

### miR-146a and miR-153 target the 3′-UTR of *BRCA1*

*BRCA1* gene expression is frequently down-regulated in triple-negative breast cancers. We hypothesized that the identification of miRNAs targeting the 3′-UTR of *BRCA1* might be used to uncover miRNAs involved in the development of triple-negative breast cancers. To this end, we performed an *in silico* computational search using five available algorithms: TargescanHuman [27], Tarbase [28], Miranda [29], MicroCosmTargets [30] and PicTar [31].

For all algorithms we used default parameters, and we selected miRNAs predicted to target *BRCA1* by at least three algorithms. From the selected miRNAs (listed in Table S4), we chose five miRNAs, i.e., two miRNAs (miR-146a and miR-146b-5p) that were previously found to be over-expressed in breast tumors and to down-regulate *BRCA1* expression [18] and three miRNAs (miR-132, miR-212, miR-153) that were most frequently predicted to interact with the 3′-UTR of *BRCA1*. We validated the expression of these five selected miRNAs by qRT-PCR in the 10 cell lines and found that miR-146a was higher expressed in 3 triple-negative breast cancer cell lines: MDA-MB-231, MDA-MB-436 and SUM1315-BRCA1 (Fig. 1c), that miR-153 was higher expressed in 4 triple-negative breast cancer cell lines: MDA-MB-436, SUM1315MO2, SUM149PT and HCC1937 (Fig. 1d), and that miR-146b-5p, miR-132 and miR-212 were expressed in all 10 cell lines at different levels (Fig. S1) without a significant difference between the triple-negative, luminal or benign cell lines. Based on these results we focused our further analyses, next to miR-10b and miR-26a, on miR-146a and miR-153.

### *BRCA1* expression is regulated by miR-10b, miR-26a and miR-153

The above expression profiling and *in silico* analyses allowed us to select 4 miRNAs, miR-10b, miR-26a, miR-146a and miR-153 for further analysis. To determine the effect of these miRNAs on endogenous *BRCA1* expression, we transfected two BRCA1 wild-type cell lines (MDA-MB-231 and MCF7) with the respective miRNAs (miRNA mimics) and anti-miRNAs (inhibitors or ‘antagomirs’). The efficiency of the miRNA mimics and inhibitors was verified by miRNA quantification using qRT-PCR (Fig. S2), revealing sufficient efficiencies only for miR-10b, miR-26a, miR-146a, anti-miR-146a and miR-153. The *BRCA1* siRNA efficiency was also tested and found to be sufficient (Fig. S3). Since MCF7 is a luminal cell line and MDA-MB-231 a triple-negative cell line, our data can potentially reveal whether the miRNAs tested may have a different effect on *BRCA1* in these two subtypes of breast cancer.

**Fig. 2:**
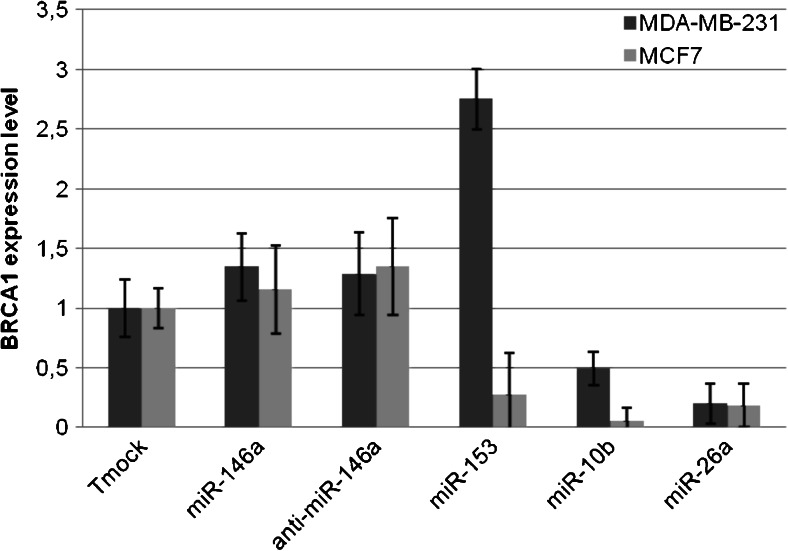
*BRCA1* expression after miRNA mimic and miRNA inhibitor transfection in MDA-MB-231 and MCF7 cells. Expression of *BRCA1* was determined by qRT-PCR in MDA-MB-231 and MCF7 cells transfected with Tmock (transfection reagent only), miR-146a, anti-miR-146a, miR-153, miR10-b and miR-26a. *BRCA1* expression was normalized using 18S

To determine whether miR-146a affects endogenous *BRCA1* expression, we compared the expression level in MDA-MB-231 and MCF7 cells after transfection with miR-146a, anti-miR-146a and a mock control (transfection reagent alone). By doing so, we found that miR-146a does not appear to regulate the expression of *BRCA1* (Fig. 2). Next, we tested the effect of miR-153 on the expression of *BRCA1*. The results obtained (Fig. 2) indicate that miR-153 can down-regulate the expression of *BRCA1* in MCF7 cells. In contrast, we found that in MDA-MB-231 cells miR-153 induced *BRCA1* up-regulation. After miR-10b transfection into MDA-MB-231 and MCF7, the *BRCA1*expression level was found to be decreased in both of them (Fig. 2). These results suggest that miR-10b can down-regulate the expression of *BRCA1* in MDA-MB-231 and MCF7 cells. After miR-26a transfection into MDA-MB-231 and MCF7 cells, the expression level of *BRCA1* was again found to be decreased in both of them (Fig. 2), indicating that also miR-26a can down-regulate the expression of *BRCA1* in MDA-MB-231 and MCF7 cells.

### *EGFR* expression is not regulated by miR-146a in MDA-MB-231 and MCF7 cells

Kumaraswamy et al. reported that *BRCA1* can induce miR-146a expression which, in turn, can repress *EGFR* expression in mammary cells [25]. To determine whether miR-146a can affect endogenous *EGFR* expression in our cell lines, we compared its expression level in MDA-MB-231 and MCF7 cells after transfection with miR-146a, anti-miR-146a, Tmock (transfection reagent alone) and siBRCA1 (Fig. S4). After doing so, we found that in these cell lines miR-146a had no effect on the expression of *EGFR*.

### Stimulation and inhibition of cell proliferation by miRNAs

**Fig. 3:**
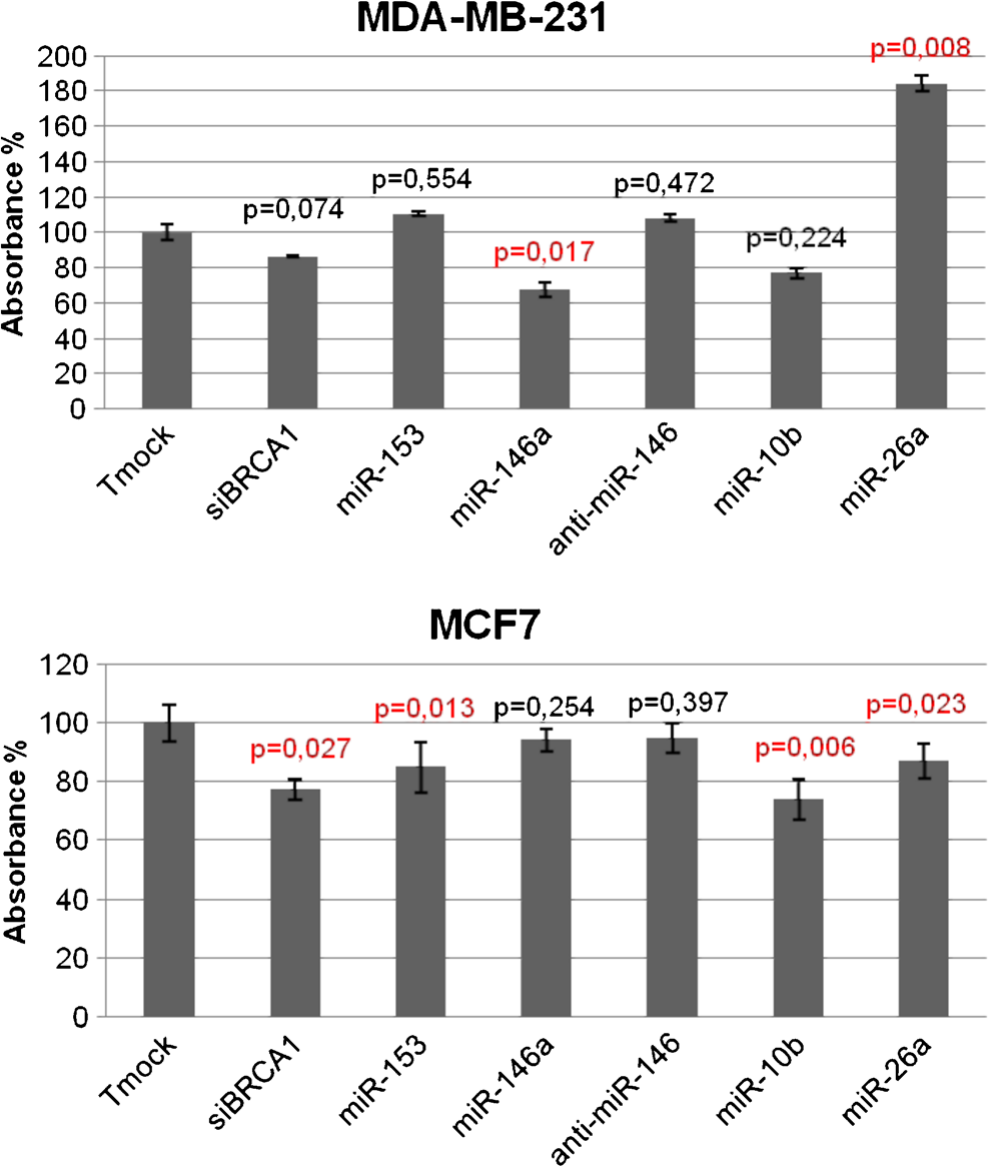
Proliferation assay after siBRCA1, miRNA mimic and inhibitor transfection in MDA-MB-231 and MCF7 cells. Cells were transfected with Tmock (transfection reagent only), siBRCA1, miR-146a, anti-miR-146, miR-153, miR-10b and miR-26a. After 48 h, *in vitro* cell proliferation was evaluated using CCK-8. The absorbance was determined at 450 nm. All experiments were performed in triplicate

To assess the effect of miR-10b, miR-26a, miR146a, anti-miR-146a and miR-153 on MDA-MB-231 and MCF7 cell proliferation, the cells were transfected with the respective (anti-) miRNAs, as also siBRCA1 and Tmock (transfection reagent alone). In MDA-MB-231 cells, the proliferation assay (see materials and methods) showed that miR-26a significantly stimulated and that miR-146a inhibited proliferation compared to mock-transfected cells. In MCF7 cells, miR-153, miR-10b and miR-26a significantly inhibited proliferation compared to mock-transfected cells (Fig. 3).

### Differential expression of miR-10b, miR-26a, miR-146a and miR-153 in TCGA

**Fig. 4:**
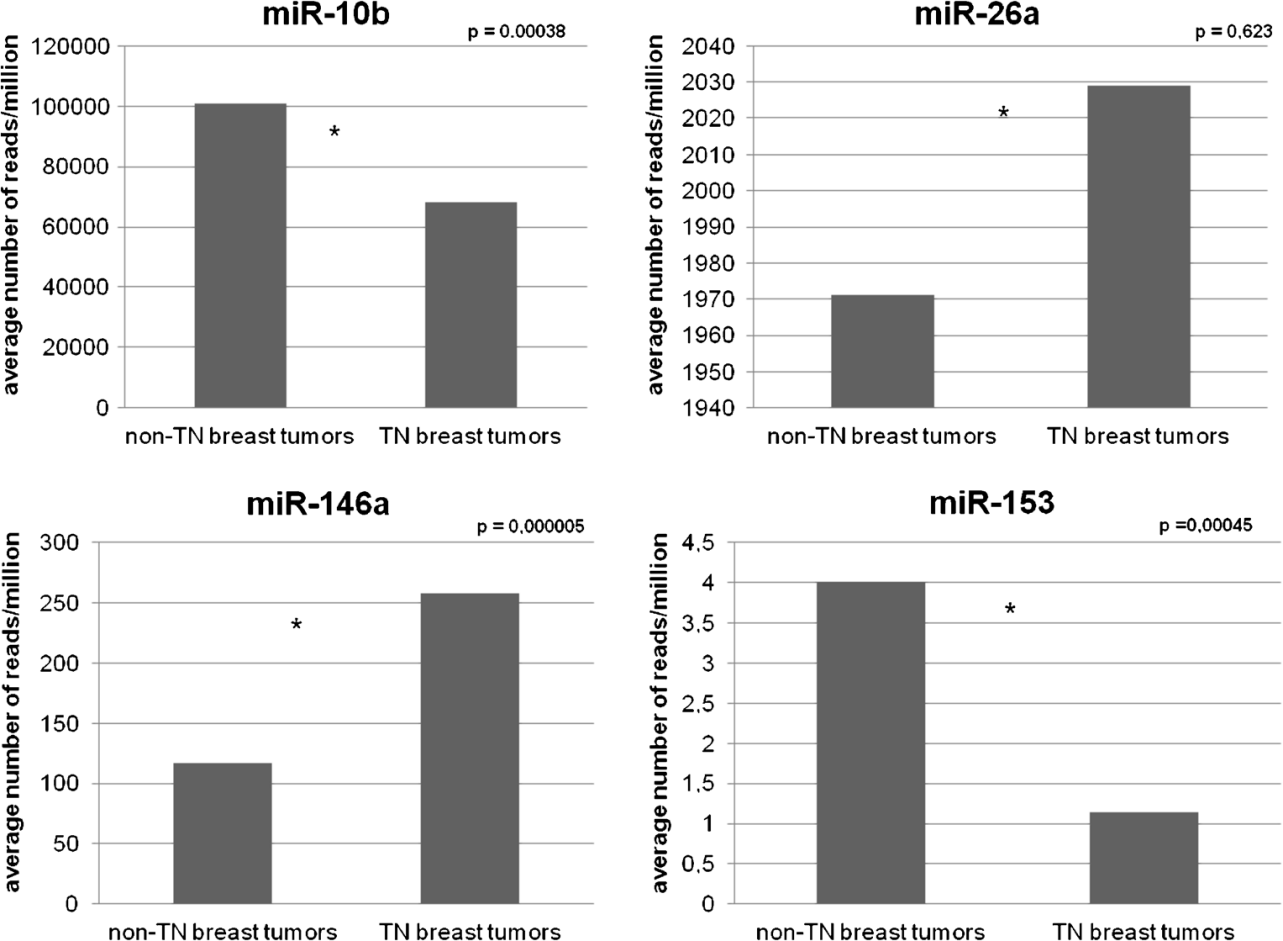
*In silico* expression analysis of miRNAs using TCGA data. Clinical and miRNA expression data for breast cancer were downloaded from The Cancer Genome Atlas (TCGA) database. The expression of four miRNAs (miR-10b, miR-26a, miR-146a and miR-153) was compared in 88 breast tumors with a negative ER, PR and HER2 status (i.e., triple negative phenotype) and in 431 breast tumors that were positive for at least one of the receptors. Student’s *t-*test was used to assess statistical differences in mean expression levels between these two groups

TCGA was queried for the expression of miR-10b, miR-26a, miR-146a and miR-153 in different primary breast cancer tissues. As shown in Fig. 4 the average expression of miR-153 and miR-10b was found to be significantly lower in triple-negative tumors (*p* = 0.00045 and *p* = 0.00038, respectively) than in luminal tumors, while the expression of miR-146a was found to be significantly higher in triple-negative tumors (*p* = 0.000005). For miR-26a, the difference in average expression was not found to be significant in these tumors (*p* = 0.6234).

## Discussion

Triple-negative breast cancers, representing ~10 to 15 % of all human breast cancers, have gained growing interest in recent years [32]. In addition, the role of microRNAs (miRNAs) in the epigenetic regulation of many cellular processes has increasingly become recognized as an important way to fine-tune gene expression [33]. Here, expression profiling, transfection and *in silico* assays were performed to identify potential triple-negative breast cancer miRNA biomarkers, i.e., miR-10b, miR-26a, miR-146a and miR-153.

We found that miR-10b was higher expressed in triple-negative breast cancer-derived cell lines compared to luminal breast cancer-derived cell lines. TCGA data, however, indicated that the average expression of miR-10b was significantly lower in triple-negative tumors than in non triple-negative tumors. O’Day and Lal reported that miR-10b was over-expressed only in metastatic cancer cells, and was found to promote tumor cell migration, invasion and metastasis *in vivo* [20]. Whereas miR-10b has been found to be highly expressed in several human cancers [34], Biagioni et al. found that miRNA-10b was down-regulated in tumor tissues compared to their normal matched counterparts due to hypermethylation of CpG islands upstream from the miR-10b/10b* locus [35]. We found that miR-10b can down-regulate the expression of *BRCA1* in MDA-MB-231 and MCF7 cells. High miR-10b expression may thus be a way to silence *BRCA1* expression in *BRCA1* wild-type cells. Recently, it was reported that miR-10b may also play a critical role in TGF-induced epithelial-mesenchymal transition (EMT) in breast cancer and, as such, may be considered as a possible therapeutic target [36]. Moreover, miR-10b was shown to be able to promote the invasion and metastasis of tumor cells through post-transcriptional regulation of *HOXD10* [37]. Overall, these results indicate that miR-10b may act as an oncogenic miRNA in breast cancer cells. Nevertheless, Biagioni et al. reported that exogenous miR-10b expression reduced both the *in vitro* and *in vivo* proliferative capacities of MCF7 and MDA-MB-468 cells [35]. Our results are compatible with those of Biagioni et al., as we found that miR-10b significantly inhibited the proliferation of MCF7 cells (*p* = 0.006). Thus, restoration of miR-10b expression may hold a therapeutic promise for breast cancer treatment.

Another potential biomarker of triple-negative breast cancer that we identified is miR-26a, and we found that miR-26a can down-regulate the expression of *BRCA1* in MDA-MB-231 and MCF7 cells. These results suggest an oncogenic function of this miRNA in breast cancer. In contrast, Gao et al. reported that miR-26a could inhibit the proliferation and migration of breast cancer cells through repression of *MCL-1*, and that miR-26a could increase the sensitivity of breast cancer cells to paclitaxel [38]. Others found that miR-26a can promote ovarian cancer cell proliferation by targeting *ERα* [39]. We found that miR-26a significantly stimulated the proliferation of MDA-MB-231 cells and inhibited the proliferation of MCF7 cells. We also found that the average expression of miR-26a was not significantly different between triple-negative and non triple-negative breast tumors in the TCGA database. The role of miR-26a thus appears to be complex and may dependent on the tissue and/or tumor context.

Our cell line and TCGA data analyses show that miR-146a is significantly over-expressed in triple-negative breast cancers. Similar results were reported by others [18, 25]. We also found that miR-146a does not affect the expression of *BRCA1* in MDA-MB-231 and MCF7 cells. In contrast, Gracia et al. reported that miR-146a can down-regulate the expression of *BRCA1* in triple-negative sporadic breast cancers [18]. This discrepancy may be due to the different cell lines used for the exogenous expression assays, i.e., Gracia et al. used HeLa cells and three mammary cell lines (MDA-MB-468, MDA-MB-157 and MDA-MB-436). Another study reported that miR-146a can inhibit the invasion and migration of the highly metastatic human breast cancer cell line MDA-MB-231 [40]. These data are in line with our results showing that miR-146a can inhibit the proliferation of MDA-MB-231 cells. In a recent study it was hypothesized that BRCA1 may down-regulate the expression of the *EGFR* gene by increasing the miR-146a level in breast cancer-derived cells (HCC1937) [25]. Our results are compatible with this hypothesis, as we found an increase in miR-146a expression in *BRCA1* wild-type SUM1315-BRCA1 cells compared to *BRCA1* mutated SUM1315-LXSN cells. We also found that exogenous expression of miR-146a decreased the proliferation of MDA-MB-231 cells that highly express *EGFR*, but not in MCF7 cells that do not express *EGFR*. qRT-PCR assessment of *EGFR* expression in exogenous miR-146a expressing MDA-MB-231 and MCF7 cells revealed that the *EGFR* gene was not significantly regulated by this miRNA. Taken together, these results suggest a complex feedback regulation between miR-146a and *BRCA1*, which again most likely depends on the cellular context.

Using bioinformatic tools, we identified a binding site for miR-153 in the 3′-UTR of *BRCA1*. Subsequently, we found that miR-153 can induce *BRCA1* down-regulation in MCF7 cells and up-regulation in MDA-MB-231 cells. These contradictory results reflect those reported in the literature. On one hand, Anaya-Ruiz et al. reported that silencing of miR-153 significantly inhibited the growth, reduced the proliferation and induced apoptosis in the triple-negative sporadic breast cancer-derived cell line MDA-MB-231. These results indicate that miR-153 may function as an oncogenic miRNA, whose deregulation could be involved in the initiation and/or development of human breast cancer [41]. In another report, Wu et al. suggested that miR-153 may play an important role in promoting the proliferation of human prostate cancer cells and, as such, may represent a novel mechanism of miRNA-mediated *PTEN* silencing in prostate cancer cells [42]. These data are compatible with our finding that *BRCA1* up-regulation by miR-153 had no effect on the proliferation of MDA-MB-231 cells. On the other hand, Zhao et al. found that transient transfection of miR-153 into glioblastoma multiforme stem cells (GBM-SCs) can inhibit their stemness properties, repress their growth potential and induce apoptosis [43]. These latter data are consistent with our results in MCF7 cells: although *BRCA1* was down-regulated by miR-153, it inhibit proliferation. This difference in response observed between MDA-MB-231 and MCF7 cells may be explained by differences in the endogenous levels of miR-153, i.e., higher in luminal MCF7 cells and lower in triple-negative MDA-MB-231 cells. TCGA data analysis also indicated that the average expression of miR-153 is significantly lower in triple-negative breast cancers. We conclude that also the function of miR-153 is complex, and identifying targets of miR-153 would be an interesting means to better understand its role in cell proliferation and carcinogenesis-related processes.

In conclusion, we identified miR-10b, miR-26a, miR-146a and miR-153 as potential triple-negative breast cancer biomarkers. These miRNAs may be instrumental for the future design of novel targeted treatment options of these breast cancers. This ‘from bench-top to bed-side’ translation remains, however, challenging [44] and additional research is required to better understand the function(s) of these miRNAs.

## Electronic supplementary material

Fig. S1. Expression level of miR-146b-5p, miR-132 and miR-212 in mammary cell lines. Expression of miR determined by qRT-PCR in ten mammary cell lines and normalized using U6 expression. (GIF 21 kb)

High resolution image (TIFF 507 kb)

Fig. S2. miRNAs (miR and anti-miR) expression levels in mammary cell lines (MDA-MB-231 and MCF7). The expression levels of miR and anti-miR were determined by qRT-PCR in two mammary cell lines. The expression levels were normalized using RNU6B (RNU6-2) expression. The result of anti-miR-153 is not presented since anti-miR-153 was not effective. Tmock was used as a mock control (transfection reagent alone). (GIF 13 kb)

High resolution image (TIFF 274 kb)

Fig. S3 *BRCA1* inhibition by siRNA. The expression level of *BRCA1* was determined by qRT-PCR in two mammary cell lines transfected with Tmock (transfection reagent alone) and siBRCA1. *BRCA1* expression was normalized using 18S expression (GIF 7 kb)

High resolution image (TIFF 128 kb)

Fig. S4 EGFR expression levels after miR-146a transfection in MDA-MB-231 and MCF7 cell lines. The expression level of EGFR was determined by qRT-PCR in two mammary cell lines transfected with Tmock (transfection reagent alone), miR-146a, anti-miR-146a and siBRCA1. The expression level was normalized using 18S expression (GIF 10 kb)

High resolution image (TIFF 189 kb)

Table S1 (DOCX 11 kb)

Table S2 (DOCX 11 kb)

Table S3 (DOCX 45 kb)

Table S4 (DOCX 13 kb)
