# Supplementary figures and images for: Identification of miR-10b, miR-26a, miR-146a and miR-153 as potential triple-negative breast cancer biomarkers
Source: Cell Oncol (Dordr). 2015 Sep 21;38(6):433–42. doi: 10.1007/s13402-015-0239-3 (PMC4653246; doi:10.1007/s13402-015-0239-3)

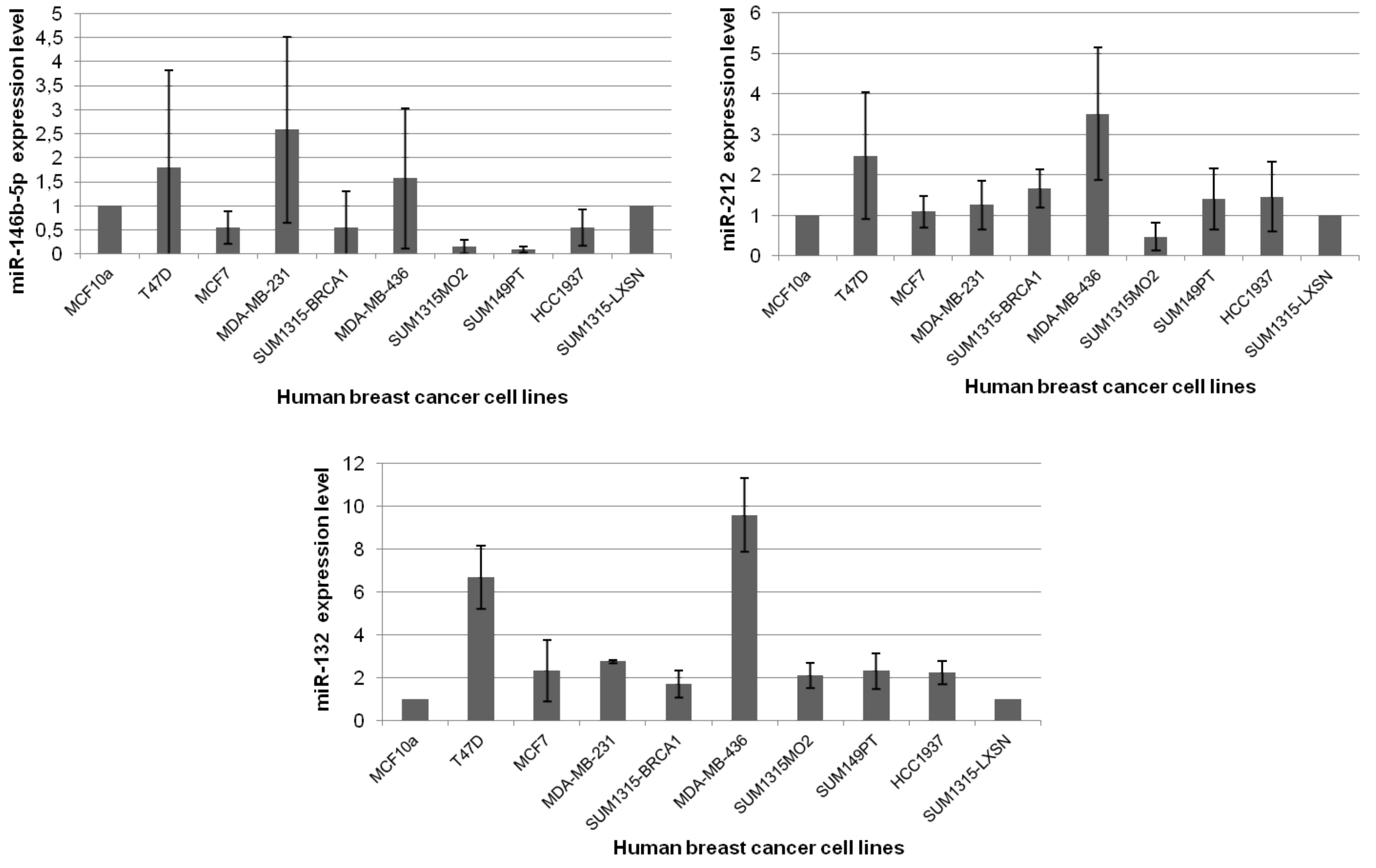

Supplement: Supplementary file 1 — Expression level of miR-146b-5p, miR-132 and miR-212 in mammary cell lines. Expression of miR determined by qRT-PCR in ten mammary cell lines and normalized using U6 expression. (GIF 21 kb) [file 13402_2015_239_Fig5_ESM.gif]

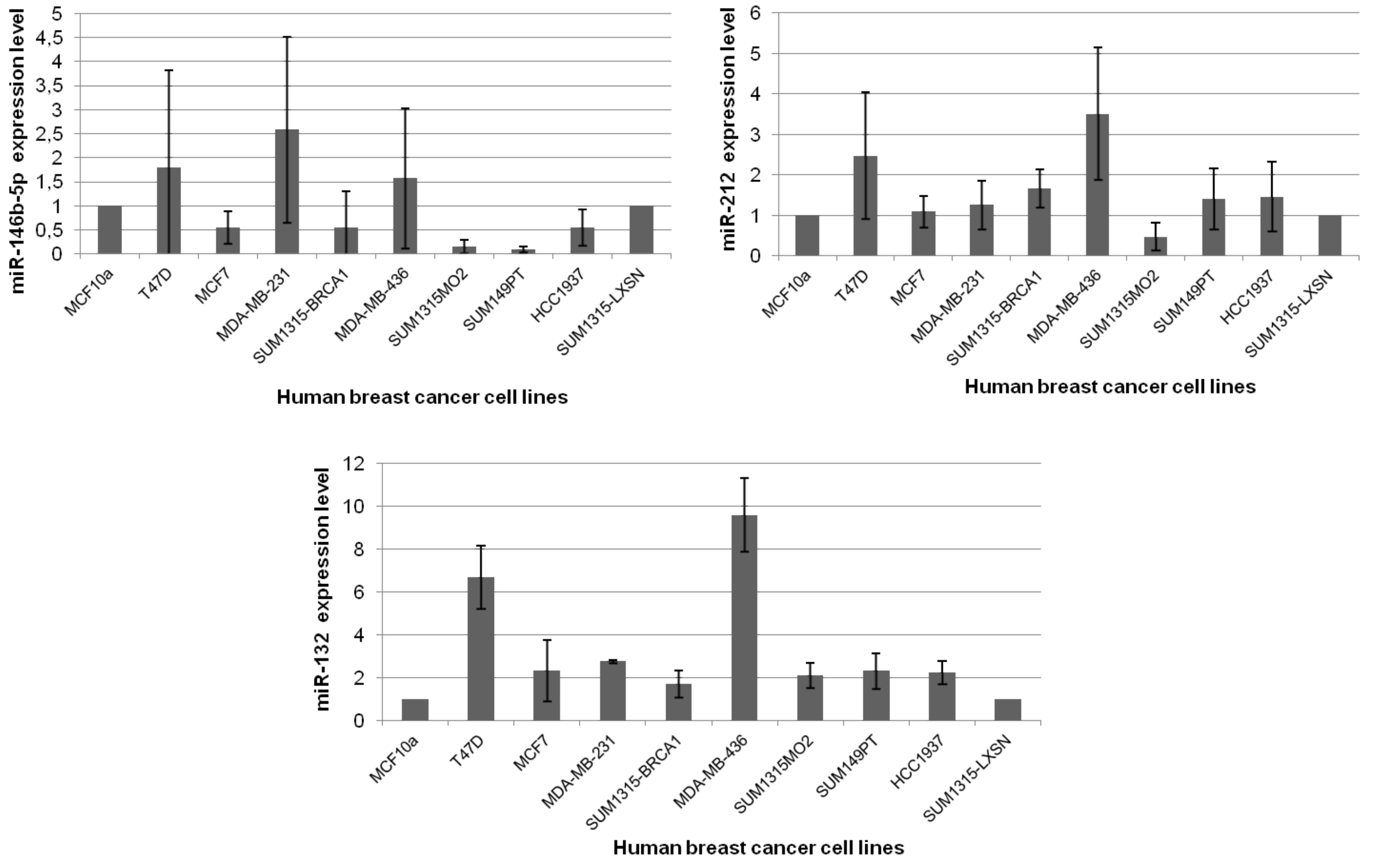

Supplement: Supplementary file 2 — High resolution image (TIFF 507 kb) [file 13402_2015_239_MOESM1_ESM.tif]

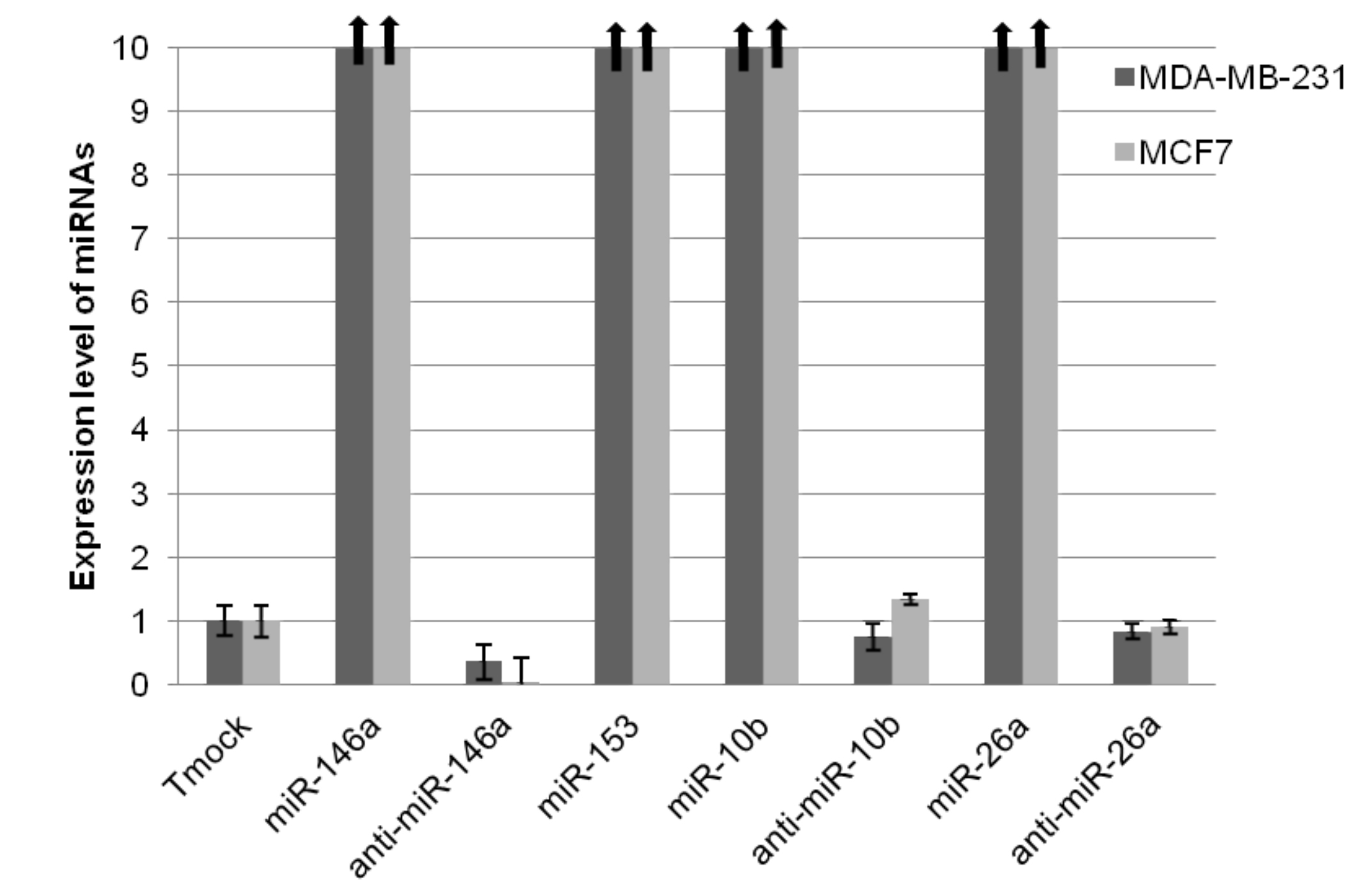

Supplement: Supplementary file 3 — miRNAs (miR and anti-miR) expression levels in mammary cell lines (MDA-MB-231 and MCF7). The expression levels of miR and anti-miR were determined by qRT-PCR in two mammary cell lines. The expression levels were normalized using RNU6B (RNU6-2) expression. The result of anti-miR-153 is not presented since anti-miR-153 was not effective. Tmock was used as a mock control (transfection reagent alone). (GIF 13 kb) [file 13402_2015_239_Fig6_ESM.gif]

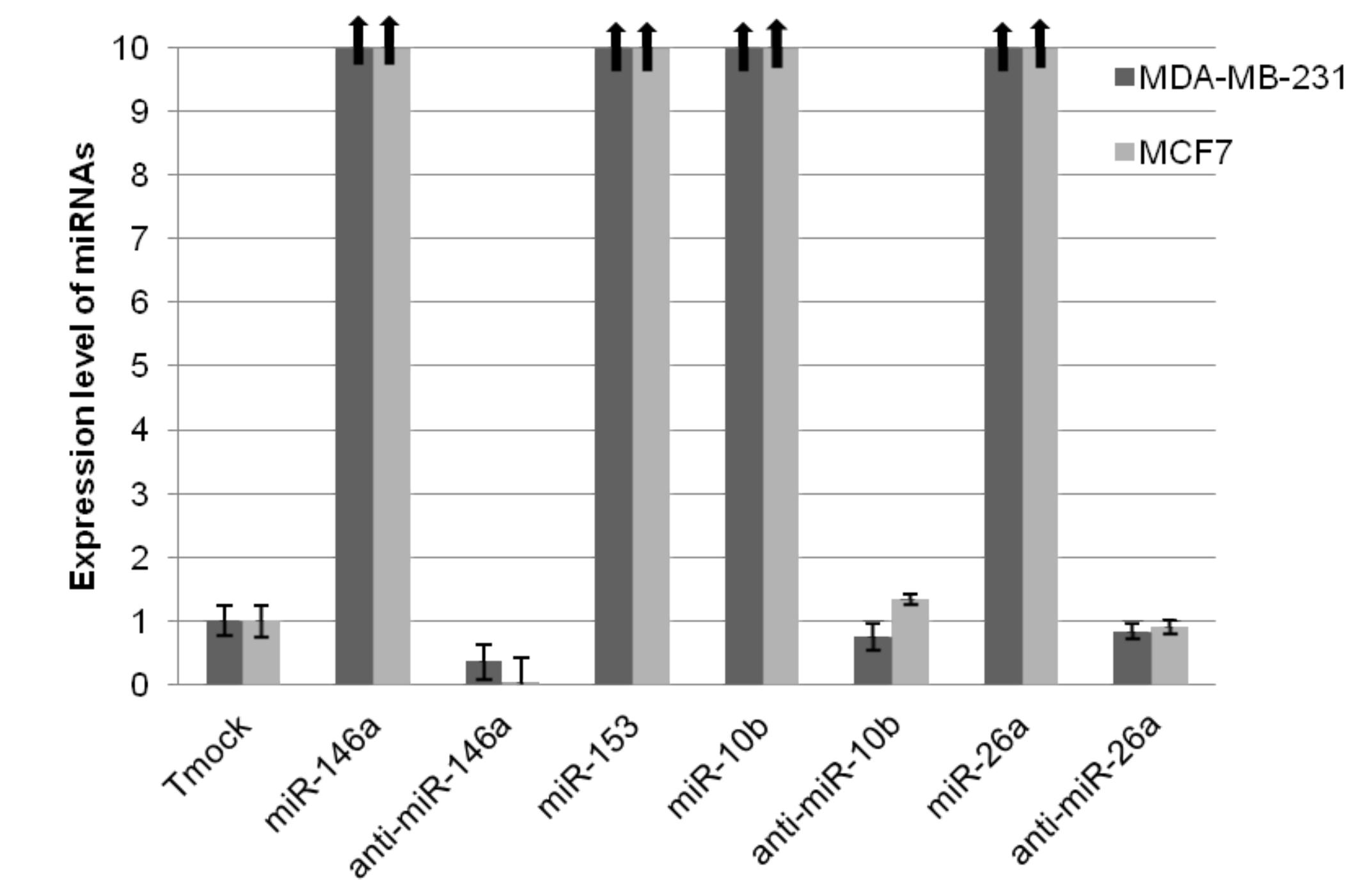

Supplement: Supplementary file 4 — High resolution image (TIFF 274 kb) [file 13402_2015_239_MOESM2_ESM.tif]

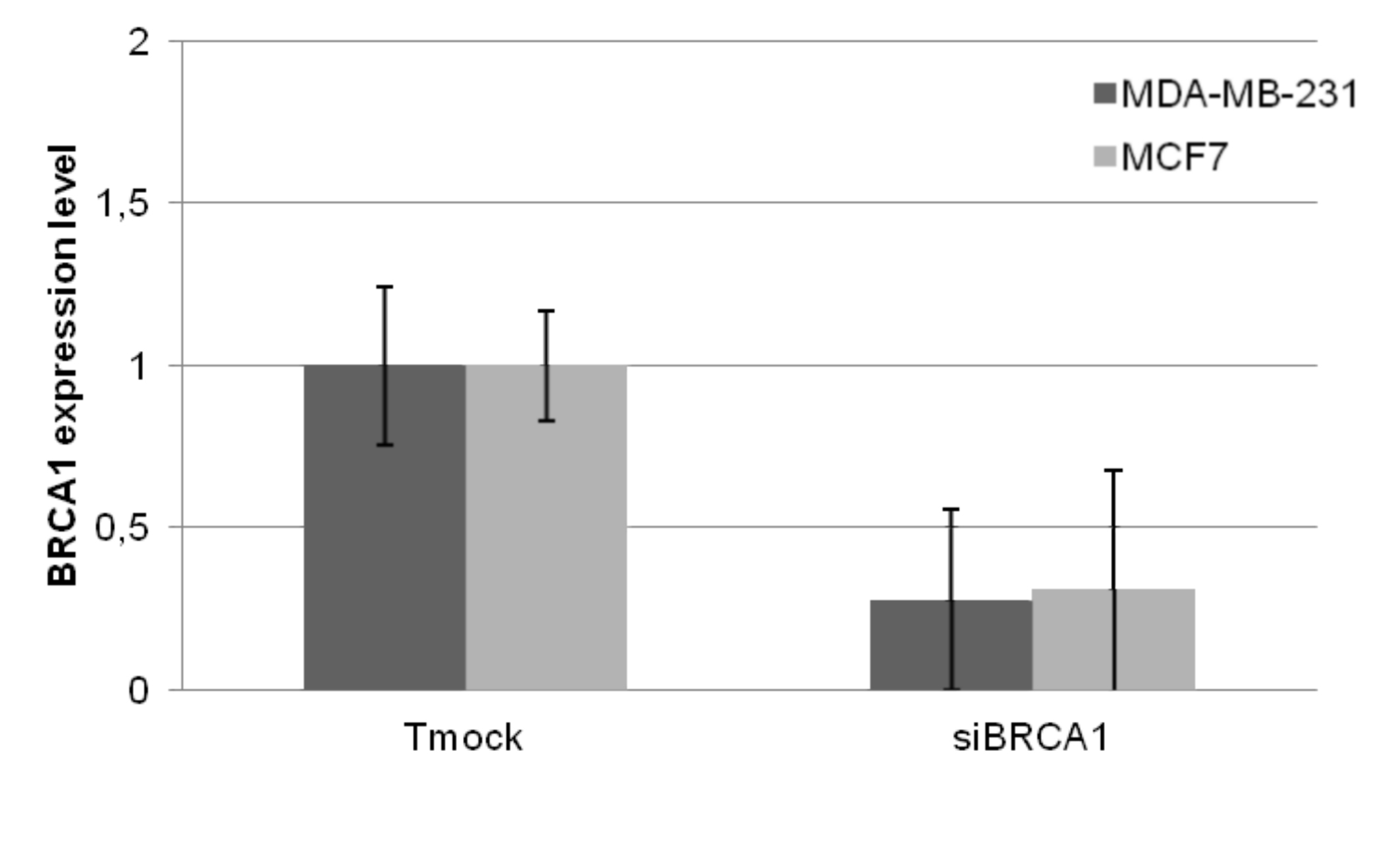

Supplement: Supplementary file 5 — BRCA1 inhibition by siRNA. The expression level of BRCA1 was determined by qRT-PCR in two mammary cell lines transfected with Tmock (transfection reagent alone) and siBRCA1. BRCA1 expression was normalized using 18S expression (GIF 7 kb) [file 13402_2015_239_Fig7_ESM.gif]

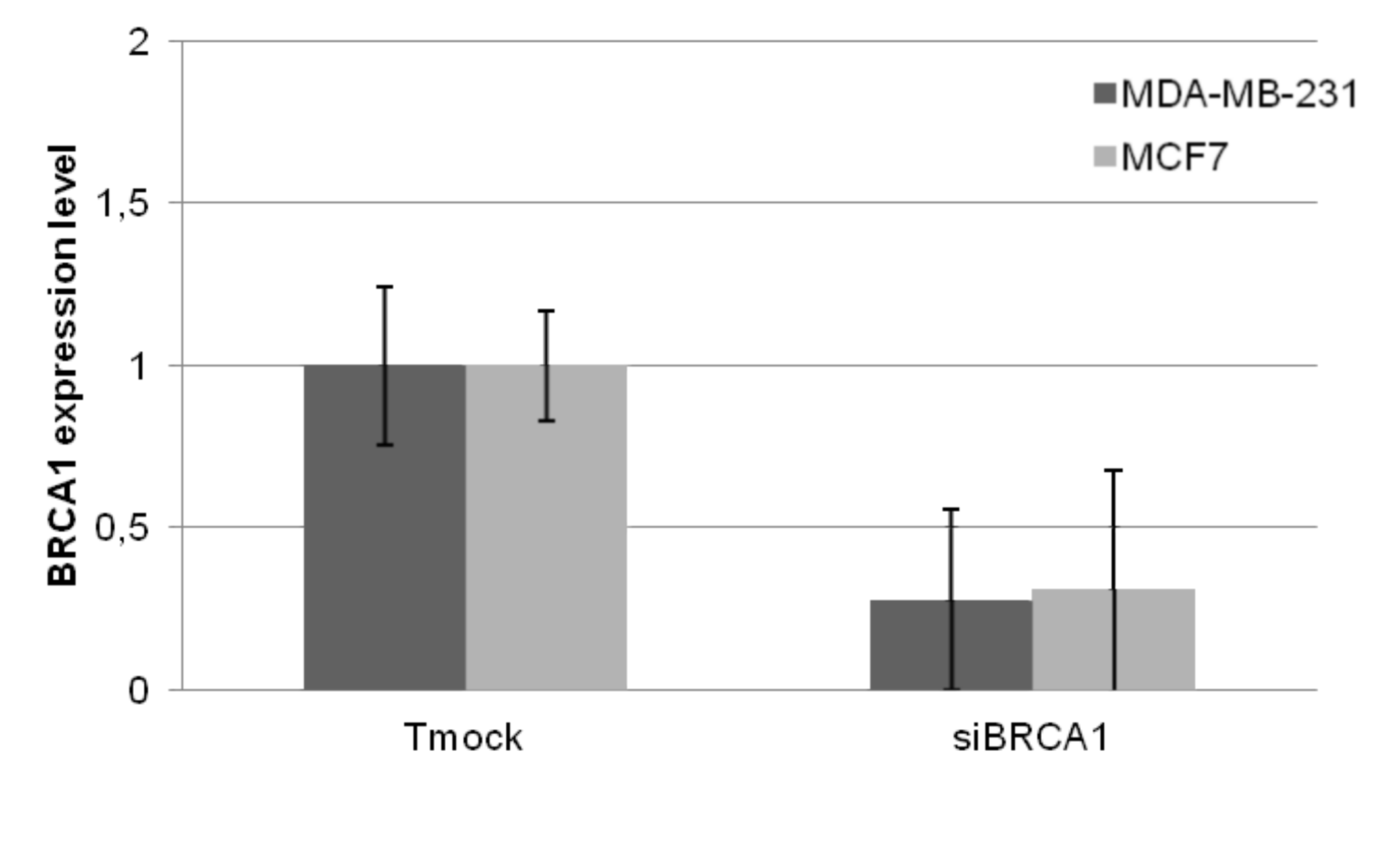

Supplement: Supplementary file 6 — High resolution image (TIFF 128 kb) [file 13402_2015_239_MOESM3_ESM.tif]

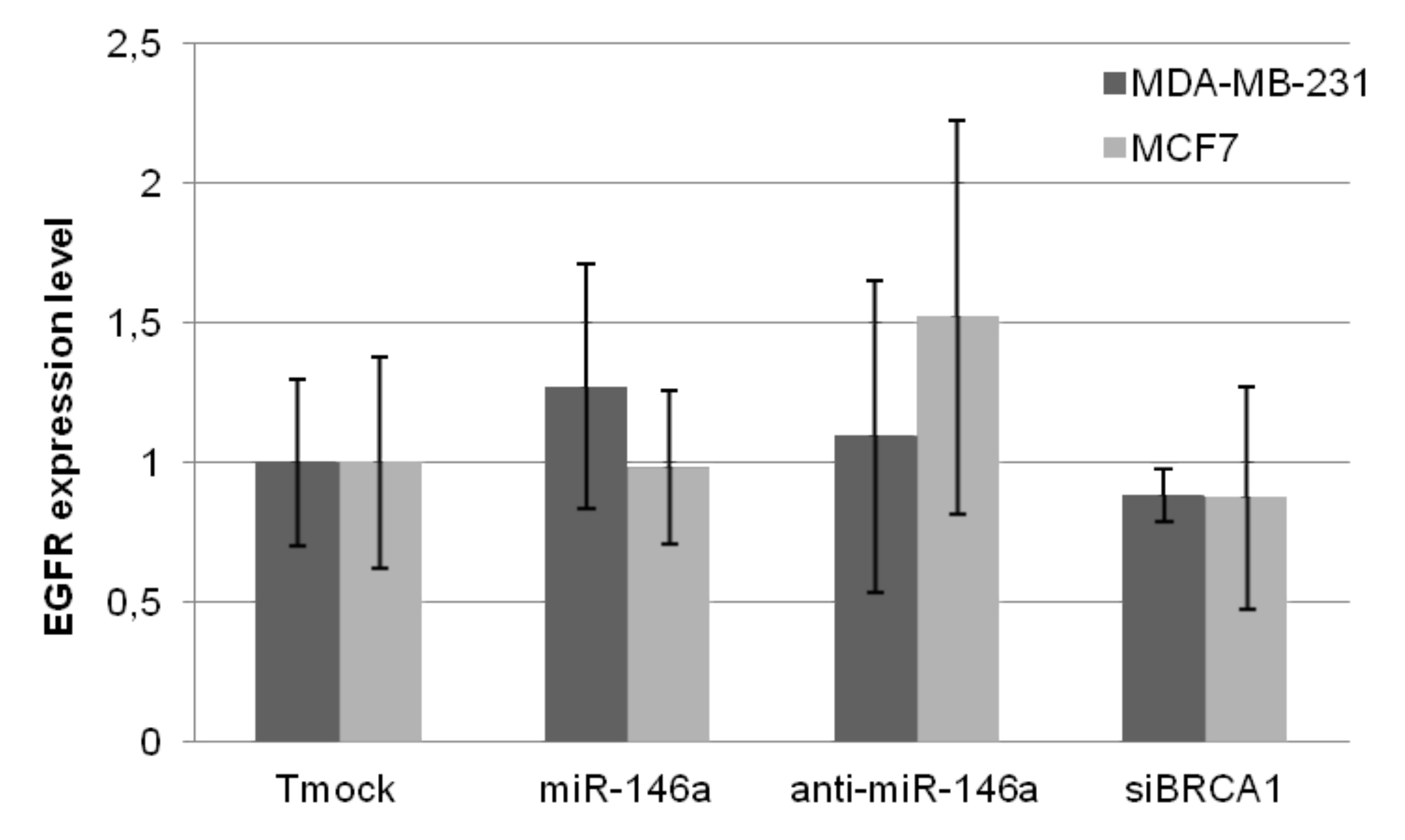

Supplement: Supplementary file 7 — EGFR expression levels after miR-146a transfection in MDA-MB-231 and MCF7 cell lines. The expression level of EGFR was determined by qRT-PCR in two mammary cell lines transfected with Tmock (transfection reagent alone), miR-146a, anti-miR-146a and siBRCA1. The expression level was normalized using 18S expression (GIF 10 kb) [file 13402_2015_239_Fig8_ESM.gif]

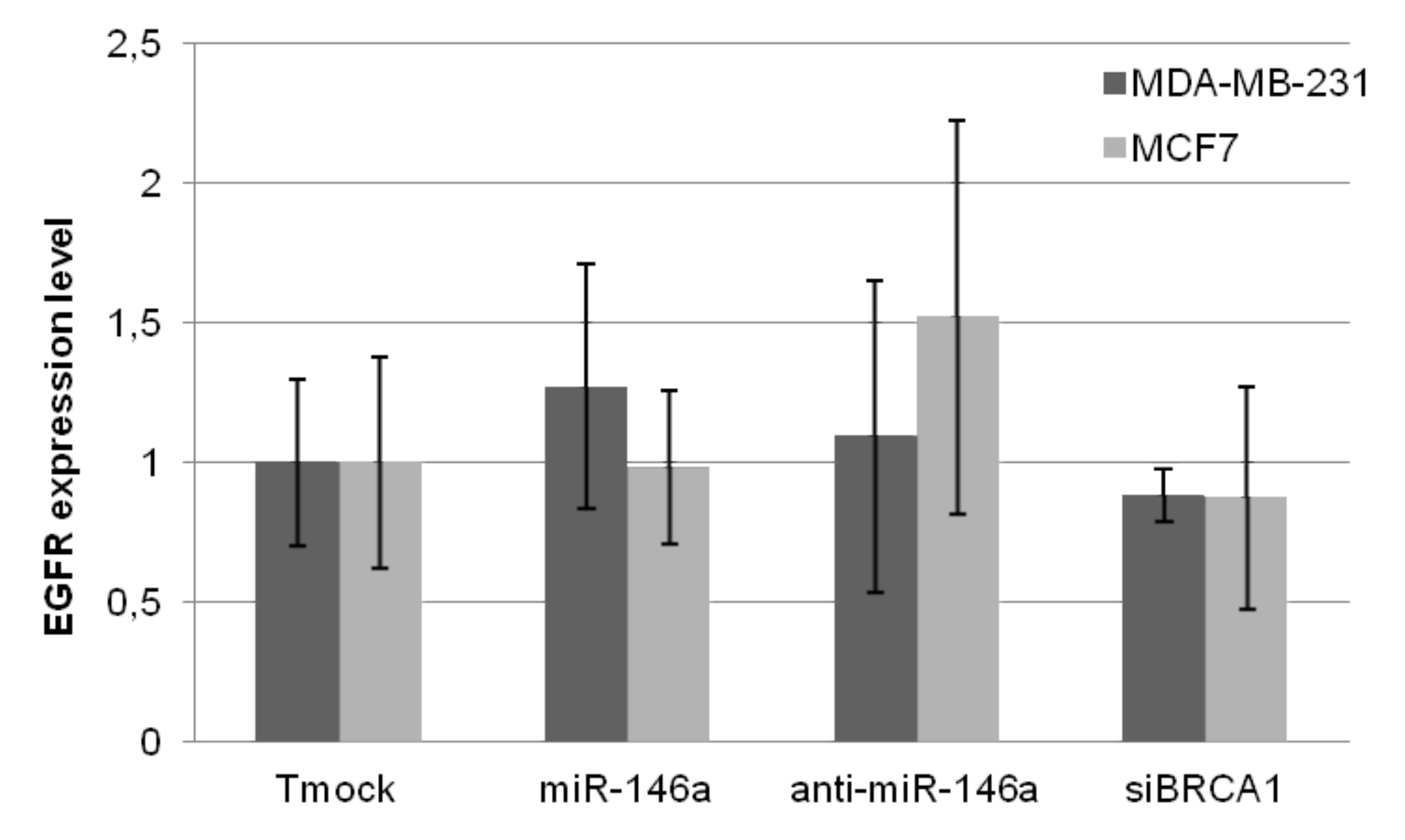

Supplement: Supplementary file 8 — High resolution image (TIFF 189 kb) [file 13402_2015_239_MOESM4_ESM.tif]
