# Supplementary material for: Identification of miR-10b, miR-26a, miR-146a and miR-153 as potential triple-negative breast cancer biomarkers
Source: Cell Oncol (Dordr). 2015 Sep 21;38(6):433–42. doi: 10.1007/s13402-015-0239-3 (PMC4653246; doi:10.1007/s13402-015-0239-3)
Supplement: Supplementary file 9 — (DOCX 11 kb) [file 13402_2015_239_MOESM5_ESM.docx]

**Table S1** List of the analyzed microRNAs

| **miScript Primer Assay** | **QIAGEN Cat No** | **Mature miRNA sequence** |
| --- | --- | --- |
| Hs_miR-146a_1 | MS00003535 | UGAGAACUGAAUUCCAUGGGUU |
| Hs_miR-146b_1 | MS00003542 | UGAGAACUGAAUUCCAUAGGCU |
| Hs_miR-153_1 | MS00008771 | UUGCAUAGUCACAAAAGUGAUC |
| Hs_miR-132_1 | MS00003458 | UAACAGUCUACAGCCAUGGUCG |
| Hs_miR-212_1 | MS00003815 | UAACAGUCUCCAGUCACGGCC |
| Hs_miR-10b_3 | MS00031269 | UACCCUGUAGAACCGAAUUUGUG |
| Hs_miR-26a_2 | MS00029239 | UUCAAGUAAUCCAGGAUAGGCU |
| Hs_miR-15b_2 | MS00008792 | UAGCAGCACAUCAUGGUUUACA |
| Hs_miR-206_1 | MS00003787 | UGGAAUGUAAGGAAGUGUGUGG |
| Hs_miR-155_2 | MS00031486 | UUAAUGCUAAUCGUGAUAGGGGU |
| Hs_miR-485-5p_1 | MS00006972 | AGAGGCUGGCCGUGAUGAAUUC |
