# Supplementary material for: Identification of miR-10b, miR-26a, miR-146a and miR-153 as potential triple-negative breast cancer biomarkers
Source: Cell Oncol (Dordr). 2015 Sep 21;38(6):433–42. doi: 10.1007/s13402-015-0239-3 (PMC4653246; doi:10.1007/s13402-015-0239-3)
Supplement: Supplementary file 10 — (DOCX 11 kb) [file 13402_2015_239_MOESM6_ESM.docx]

**Table S2** List of miRNA mimics and miRNA inhibitor

| **Assay name** | **QIAGEN Cat No** | **Mature miRNA sequence** |
| --- | --- | --- |
| Syn-hsa-miR-146a-5p  miScript miRNA Mimic | MSY0000449 | 5' GAGAACUGAAUUCCAUGGGUU |
| Anti-hsa-miR-146a-5p  miScript miRNA Inhibitor | MIN0000449 | 5' GAGAACUGAAUUCCAUGGGUU |
| Syn-hsa-miR-153  miScript miRNA Mimic | MSY0000439 | 5' UGCAUAGUCACAAAAGUGAUC |
| Anti-hsa-miR-153  miScript miRNA Inhibitor | MIN0000439 | 5' UGCAUAGUCACAAAAGUGAUC |
| Syn-hsa-miR-10b-5p  miScript miRNA Mimic | MSY0000254 | 5'UACCCUGUAGAACCGAAUUUGUG |
| Anti-hsa-miR-10b-5p  miScript miRNA Inhibitor | MIN0000254 | 5'UACCCUGUAGAACCGAAUUUGUG |
| Syn-hsa-miR-26a-5p  miScript miRNA Mimic | MSY0000082 | 5' UUCAAGUAAUCCAGGAUAGGCU |
| Anti-hsa-miR-26a-5p  miScript miRNA Inhibitor | MIN0000082 | 5' UUCAAGUAAUCCAGGAUAGGCU |
