# Supplementary material for: Identification of miR-10b, miR-26a, miR-146a and miR-153 as potential triple-negative breast cancer biomarkers
Source: Cell Oncol (Dordr). 2015 Sep 21;38(6):433–42. doi: 10.1007/s13402-015-0239-3 (PMC4653246; doi:10.1007/s13402-015-0239-3)
Supplement: Supplementary file 11 — (DOCX 45 kb) [file 13402_2015_239_MOESM7_ESM.docx]

**Table S3** Profiling of microRNAs involved in human breast cancer cell lines by miScript miRNA PCR Array when comparing subgroups of cell lines

| **p**  **o**  **s**  **i**  **t**  **i**  **o**  **n** | **miRNA** | **SUM1315-LXSN / SUM1315-BRCA1** | | **Basal BRCA1+/ Luminal** | | **Basal BRCA1- / Luminal** | | **Tumoral / Benign** | | **Basal BRCA1- / Benign** | | **Basal BRCA1+ / Benign** | |
| --- | --- | --- | --- | --- | --- | --- | --- | --- | --- | --- | --- | --- | --- |
|  |  | **P-value** | **Fold Change** | **P-value** | **Fold Change** | **P-value** | **Fold Change** | **P-value** | **Fold Change** | **P-value** | **Fold Change** | **P-value** | **Fold Change** |
| A01 | hsa-let-7a | 0.469253 | 1.2454 | 0.451748 | 1.1716 | 0.465739 | 1.1461 | 0.332273 | 1.2693 | 0.271989 | 1.398 | 0.342044 | 1.2768 |
| A02 | hsa-let-7b | 0.400036 | 1.4581 | 0.276644 | 1.8753 | 0.011316 | 2.3619 | 0.049674 | 0.6215 | 0.00895 | 0.7217 | 0.146702 | 0.6051 |
| A03 | hsa-let-7c | 0.131373 | 1.7569 | 0.273363 | 1.5853 | 0.033661 | 2.5104 | 0.171181 | 0.588 | 0.415024 | 0.7358 | 0.097511 | 0.5356 |
| A04 | hsa-let-7d | 0.519456 | 1.3574 | 0.05843 | 7.0823 | 0.08087 | 6.1314 | 0.813039 | 0.7335 | 0.78608 | 0.924 | 0.434712 | 1.3368 |
| A05 | hsa-let-7e | 0.832377 | 1.09 | 0.001074 | 0.2282 | 0.720027 | 0.4832 | 0.494518 | 1.5286 | 0.441067 | 1.8101 | 0.876171 | 0.7366 |
| A06 | hsa-let-7f | 0.754349 | 1.1444 | 0.819956 | 6.4894 | 0.681191 | 5.883 | 0.049675 | 0.1797 | 0.068761 | 0.1911 | 0.079019 | 0.2821 |
| A07 | hsa-let-7g | 0.401202 | 0.4536 | 0.107909 | 2.7472 | 0.383246 | 1.5082 | 0.261246 | 2.3501 | 0.260056 | 2.9129 | 0.133258 | 3.6023 |
| A08 | hsa-let-7i | 0.220154 | 0.3626 | 0.307287 | 2.6645 | 0.623206 | 0.7735 | 0.61639 | 0.7509 | 0.727939 | 0.4269 | 0.338909 | 1.9028 |
| A09 | hsa-miR-1 | 0.234795 | 0.2913 | 0.454356 | 3.9889 | 0.734902 | 3.4055 | 0.478102 | 0.3366 | 0.615838 | 0.368 | 0.442542 | 0.6344 |
| A10 | hsa-miR-100 | 0.050458 | 0.3506 | 0.001755 | 6.8781 | 0.089255 | 2.3532 | 0.083607 | 3.9718 | 0.079619 | 4.156 | 0.016105 | 10.7799 |
| A11 | hsa-miR-107 | 0.088762 | 0.4442 | 0.29817 | 0.6287 | 0.207663 | 0.5752 | 0.936822 | 0.6421 | 0.706626 | 0.5518 | 0.585885 | 0.5886 |
| A12 | hsa-miR-10a | 0.07997 | 0.2722 | 0.940159 | 3.4479 | 0.852 | 2.612 | 0.73643 | 0.6049 | 0.817643 | 0.6805 | 0.805493 | 0.8916 |
| B01 | hsa-miR-10b | 0.011425 | 3.2233 | 0.007324 | 12.9602 | 0.101812 | 8.793 | 0.362882 | 3.4673 | 0.287624 | 4.8273 | 0.051559 | 9.5911 |
| B02 | hsa-miR-125b | 0.639155 | 0.958 | 0.401507 | 27.6063 | 0.06997 | 49.389 | 0.115709 | 0.2446 | 0.426421 | 0.6752 | 0.000497 | 0.3244 |
| B03 | hsa-miR-125b-1* | 0.194125 | 1.5046 | 0.493866 | 0.8862 | 0.970605 | 1.036 | 0.984631 | 0.9589 | 0.762864 | 0.9128 | 0.627365 | 0.8769 |
| B04 | hsa-miR-128 | 0.196314 | 2.1778 | 0.198812 | 0.6192 | 0.674585 | 1.1542 | 0.502332 | 0.597 | 0.706964 | 0.6055 | 0.013267 | 0.377 |
| B05 | hsa-miR-129-5p | 0.034735 | 3.5341 | 0.439516 | 1.3659 | 0.251175 | 1.7661 | 0.214975 | 1.9479 | 0.267896 | 1.9406 | 0.22106 | 2.0826 |
| B06 | hsa-miR-130a | 0.711652 | 1.2636 | 0.064341 | 0.1058 | 0.447349 | 0.2819 | 0.416946 | 7.2707 | 0.47711 | 5.1394 | 0.381591 | 2.7698 |
| B07 | hsa-miR-130b | 0.137661 | 0.4231 | 0.388706 | 2.5094 | 0.599461 | 0.2927 | 0.391644 | 88.1397 | 0.480983 | 31.9807 | 0.370133 | 281.5547 |
| B08 | hsa-miR-132 | 0.406709 | 0.4408 | 0.132892 | 0.5987 | 0.136735 | 1.4989 | 0.392595 | 0.2986 | 0.518551 | 0.3246 | 0.54822 | 0.1944 |
| B09 | hsa-miR-140-5p | 0.206494 | 0.245 | 0.22215 | 5.173 | 0.256613 | 1.4842 | 0.0091 | 0.0462 | 0 | 0.0281 | 0.274647 | 0.1439 |
| B10 | hsa-miR-141 | 0.383899 | 0.2326 | 0.150868 | 79.4607 | 0.330206 | 9.3667 | 0.158092 | 5.5684 | 0.149696 | 8.9658 | 0.186156 | 43.9233 |
| B11 | hsa-miR-145 | 0.336048 | 0.6805 | 0.222411 | 3.6652 | 0.059637 | 6.5288 | 0.895238 | 0.1456 | 0.54594 | 0.2072 | 0.780475 | 0.1469 |
| B12 | hsa-miR-148a | 0.083871 | 0.2138 | 0.669094 | 7.8709 | 0.225063 | 1.0125 | 0.144133 | 11.5739 | 0.22712 | 8.2421 | 0.033442 | 54.5418 |
| C01 | hsa-miR-152 | 0.906046 | 1.2645 | 0.330944 | 0.2451 | 0.423747 | 0.5771 | 0.302759 | 0.0572 | 0.182401 | 0.0459 | 0.143721 | 0.0323 |
| C02 | hsa-miR-155 | 0.762532 | 1.1446 | 0.823646 | 1.1223 | 0.155566 | 1.5881 | 0.0008 | 0.404 | 0.010293 | 0.4489 | 0.002207 | 0.3286 |
| C03 | hsa-miR-15a | 0.152132 | 0.5705 | 0.56079 | 0.7811 | 0.113665 | 0.3536 | 0.345066 | 0.4777 | 0.180679 | 0.2869 | 0.521669 | 0.7282 |
| C04 | hsa-miR-15b | 0.283092 | 0.6381 | 0.365442 | 0.6663 | 0.002104 | 0.3133 | 0.188548 | 0.4498 | 0.006806 | 0.2578 | 0.47072 | 0.6561 |
| C05 | hsa-miR-16 | 0.021205 | 0.4883 | 0.249481 | 0.7741 | 0.018619 | 0.389 | 0.579982 | 0.6476 | 0.209927 | 0.429 | 0.987053 | 0.9484 |
| C06 | hsa-miR-17 | 0.460655 | 1.3071 | 0.483963 | 0.8794 | 0.493439 | 0.6375 | 0.358817 | 1.845 | 0.538589 | 1.0944 | 0.363264 | 2.6012 |
| C07 | hsa-miR-181a | 0.154729 | 0.29 | 0.282546 | 3.6491 | 0.027765 | 0.3799 | 0.536072 | 1.878 | 0.712017 | 0.6481 | 0.356875 | 6.2744 |
| C08 | hsa-miR-181b | 0.291721 | 0.2439 | 0.368888 | 1.6382 | 0.884094 | 0.7124 | 0.467689 | 0.9326 | 0.752849 | 0.5973 | 0.340852 | 1.725 |
| C09 | hsa-miR-181c | 0.370257 | 0.3666 | 0.325741 | 1.9559 | 0.603413 | 2.2121 | 0.10154 | 110.3344 | 0.091344 | 332.4333 | 0.208147 | 84.0685 |
| C10 | hsa-miR-181d | 0.601881 | 0.6408 | 0.360575 | 0.2221 | 0.233516 | 1.986 | 0.850268 | 0.0242 | 0.469693 | 0.0235 | 0.601622 | 0.0051 |
| C11 | hsa-miR-182 | 0.478556 | 0.5725 | 0.416928 | 0.9716 | 0.60816 | 0.8693 | 0.96461 | 0.5391 | 0.869506 | 0.4442 | 0.5223 | 0.5668 |
| C12 | hsa-miR-186 | 0.461058 | 0.6497 | 0.42272 | 0.541 | 0.810164 | 0.6754 | 0.934304 | 0.5632 | 0.969198 | 0.4777 | 0.621338 | 0.4342 |
| D01 | hsa-miR-18a | 0.540006 | 1.0865 | 0.449859 | 3.6853 | 0.4221 | 1.6177 | 0.234719 | 124.9598 | 0.256128 | 157.7433 | 0.289658 | 208.0168 |
| D02 | hsa-miR-193b | 0.390447 | 1.3069 | 0.623935 | 1.1859 | 0.048065 | 2.0229 | 0.02047 | 0.4637 | 0.126616 | 0.5759 | 0.001132 | 0.354 |
| D03 | hsa-miR-195 | 0.499477 | 1.3143 | 0.470529 | 1.2456 | 0.036352 | 2.591 | 0.549694 | 0.6518 | 0.87054 | 0.908 | 0.010839 | 0.4262 |
| D04 | hsa-miR-199b-3p | 0.11151 | 2.2692 | 0.010171 | 0.0018 | 0.077262 | 0.0095 | 0.545812 | 0.0331 | 0.467835 | 0.0161 | 0.000012 | 0.002 |
| D05 | hsa-miR-199a-5p | 0.617542 | 1.6453 | 0.00413 | 0.0031 | 0.23485 | 0.015 | 0.552305 | 0.0458 | 0.647071 | 0.0241 | 0.000001 | 0.0032 |
| D06 | hsa-miR-19a | 0.590382 | 1.3931 | 0.002482 | 0.0223 | 0.577777 | 0.1175 | 0.913586 | 0.2124 | 0.815983 | 0.1439 | 0.000235 | 0.032 |
| D07 | hsa-miR-19b | 0.474861 | 0.46 | 0.684759 | 0.7242 | 0.698541 | 0.115 | 0.404637 | 56.8953 | 0.481029 | 28.0364 | 0.372187 | 93.2699 |
| D08 | hsa-miR-200a | 0.063425 | 0.6542 | 0.059845 | 0.0109 | 0.013379 | 0.0593 | 0.500598 | 14.8467 | 0.479197 | 14.067 | 0.10352 | 1.6625 |
| D09 | hsa-miR-200b | 0.43229 | 0.9022 | 0.013378 | 0.0251 | 0.042209 | 0.0317 | 0.287583 | 0.7927 | 0.390843 | 0.19 | 0.569904 | 0.2581 |
| D10 | hsa-miR-200c | 0.407907 | 1.0381 | 0.491997 | 0.281 | 0.417578 | 0.4298 | 0.938565 | 0.2497 | 0.725703 | 0.1586 | 0.746001 | 0.1396 |
| D11 | hsa-miR-202 | 0.483687 | 0.4866 | 0.27315 | 2.8408 | 0.596867 | 1.4596 | 0.161982 | 134.6975 | 0.183105 | 240.3853 | 0.122913 | 194.3654 |
| D12 | hsa-miR-203 | 0.010841 | 0.5723 | 0.045323 | 0.2936 | 0.517595 | 0.7677 | 0.507851 | 3.3503 | 0.448069 | 4.4262 | 0.226658 | 1.6181 |
| E01 | hsa-miR-204 | 0.227354 | 0.5228 | 0.371369 | 1.3492 | 0.44212 | 2.0208 | 0.479526 | 0.0039 | 0.113713 | 0.0019 | 0.955669 | 0.0033 |
| E02 | hsa-miR-205 | 0.140759 | 1.7041 | 0.257555 | 1.7457 | 0.067869 | 2.9439 | 0.23335 | 0.477 | 0.593337 | 0.6343 | 0.011241 | 0.4268 |
| E03 | hsa-miR-206 | 0.014389 | 4.4807 | 0.001196 | 0.3735 | 0.986186 | 0.8074 | 0.47136 | 0.7013 | 0.581031 | 0.6964 | 0.001707 | 0.3929 |
| E04 | hsa-miR-20a | 0.4298 | 1.1991 | 0.014781 | 0.3414 | 0.947259 | 0.7457 | 0.685477 | 0.5269 | 0.952524 | 0.5869 | 0.000064 | 0.2448 |
| E05 | hsa-miR-20b | 0.264255 | 1.4673 | 0.011795 | 0.3422 | 0.416338 | 0.5973 | 0.184409 | 1.7482 | 0.290386 | 1.677 | 0.550439 | 1.1021 |
| E06 | hsa-miR-21 | 0.763083 | 1.0273 | 0.293824 | 6.6427 | 0.369923 | 1.2406 | 0.144538 | 62.6266 | 0.139161 | 100.6191 | 0.133975 | 192.9737 |
| E07 | hsa-miR-210 | 0.114294 | 0.245 | 0.013935 | 40.4232 | 0.019922 | 11.1123 | 0.522755 | 0.2719 | 0.012176 | 0.2966 | 0.436758 | 1.2335 |
| E08 | hsa-miR-212 | 0.133809 | 0.0001 | 0.241133 | 261592 | 0.126867 | 58.7772 | 0.588194 | 1927.7515 | 0.172775 | 882.3522 | 0.373531 | 1410341 |
| E09 | hsa-miR-214 | 0.146047 | 0.3618 | 0.060457 | 0.0788 | 0.42215 | 0.3051 | 0.586949 | 0.7613 | 0.481683 | 0.8375 | 0.001095 | 0.1447 |
| E10 | hsa-miR-22 | 0.106475 | 0.5304 | 0.261676 | 1.018 | 0.964339 | 0.0733 | 0.539003 | 56.7584 | 0.481413 | 14.5349 | 0.373897 | 128.7592 |
| E11 | hsa-miR-222 | 0.466236 | 0.5721 | 0.981617 | 0.2082 | 0.623623 | 1.2687 | 0.2485 | 0.0011 | 0.418722 | 0.0007 | 0.120749 | 0.0003 |
| E12 | hsa-miR-223 | 0.073881 | 0.4282 | 0.03811 | 2.338 | 0.318406 | 1.4496 | 0.373951 | 1.4428 | 0.36384 | 1.5862 | 0.140318 | 2.029 |
| F01 | hsa-miR-25 | 0.644243 | 1.3701 | 0.002985 | 0.1634 | 0.001676 | 0.2936 | 0.215991 | 0.4249 | 0.029041 | 0.3478 | 0.00033 | 0.1878 |
| F02 | hsa-miR-26a | 0.135969 | 1.8372 | 0.001667 | 7.6693 | 0.004885 | 10.0705 | 0.015589 | 0.3296 | 0.043122 | 0.5218 | 0.012046 | 0.466 |
| F03 | hsa-miR-26b | 0.10626 | 1.9566 | 0.28623 | 1.7335 | 0.032691 | 2.9249 | 0.004468 | 0.3604 | 0.04033 | 0.4647 | 0.001027 | 0.2984 |
| F04 | hsa-miR-27a | 0.09464 | 2.2183 | 0.293603 | 1.1245 | 0.492191 | 1.6024 | 0.106726 | 0.4236 | 0.012294 | 0.4431 | 0.007305 | 0.3622 |
| F05 | hsa-miR-27b | 0.142492 | 1.6041 | 0.062118 | 218.2051 | 0.021747 | 1718.6649 | 0.830538 | 0.037 | 0.491623 | 0.354 | 0.840375 | 0.066 |
| F06 | hsa-miR-29a | 0.833361 | 0.7 | 0.34789 | 0.7113 | 0.983621 | 1.7416 | 0.138083 | 0.0401 | 0.238396 | 0.0425 | 0.023089 | 0.0283 |
| F07 | hsa-miR-29b | 0.101773 | 0.5291 | 0.103239 | 0.416 | 0.033075 | 0.6055 | 0.86313 | 0.4644 | 0.614604 | 0.4702 | 0.165825 | 0.3098 |
| F08 | hsa-miR-29c | 0.614969 | 0.0236 | 0.580236 | 0.7927 | 0.760509 | 0.2494 | 0.04403 | 0.023 | 0.008878 | 0.0078 | 0.315031 | 0.0545 |
| F09 | hsa-miR-31 | 0.000931 | 0.4987 | 0.087094 | 9.1877 | 0.012151 | 13.1578 | 0.562748 | 0.5057 | 0.482316 | 1.1053 | 0.574516 | 0.7326 |
| F10 | hsa-miR-328 | 0.004233 | 0.5144 | 0.005365 | 0.3768 | 0.000036 | 0.2827 | 0.30473 | 1.6477 | 0.357346 | 1.1756 | 0.040355 | 1.4693 |
| F11 | hsa-miR-340 | 0.567209 | 0.6311 | 0.619011 | 0.6189 | 0.745333 | 0.6703 | 0.75512 | 0.4068 | 0.994503 | 0.2647 | 0.672312 | 0.3672 |
| F12 | hsa-miR-424 | 0.340829 | 0.3507 | 0.057056 | 3.9618 | 0.133012 | 1.761 | 0.145946 | 4.4278 | 0.096081 | 5.8301 | 0.127699 | 8.3036 |
| G01 | hsa-miR-429 | 0.300652 | 0.6137 | 0.000134 | 0.0648 | 0.40497 | 0.099 | 0.456907 | 0.8243 | 0.575216 | 0.4005 | 0.46761 | 0.2519 |
| G02 | hsa-miR-485-5p | 0.7366 | 0.9263 | 0.004716 | 0.3224 | 0.026302 | 0.4658 | 0.039434 | 3.4267 | 0.049025 | 3.1811 | 0.149457 | 1.8099 |
| G03 | hsa-miR-489 | 0.36887 | 0.7306 | 0.043032 | 0.6521 | 0.000541 | 0.5161 | 0.327518 | 0.7269 | 0.016185 | 0.5595 | 0.405062 | 0.7764 |
| G04 | hsa-miR-495 | 0.93886 | 1.3248 | 0.842543 | 0.7061 | 0.306362 | 0.5014 | 0.151699 | 0.4597 | 0.012346 | 0.2955 | 0.499997 | 0.5347 |
| G05 | hsa-miR-497 | 0.091555 | 2.5543 | 0.000028 | 0.2295 | 0.797524 | 0.5693 | 0.380853 | 1.7364 | 0.37718 | 1.6757 | 0.460118 | 0.7286 |
| G06 | hsa-miR-548c-3p | 0.622605 | 1.1746 | 0.025928 | 0.0271 | 0.00254 | 0.0324 | 0.389968 | 0.9443 | 0.228851 | 0.8399 | 0.520505 | 0.2845 |
| G07 | hsa-miR-607 | 0.006152 | 0.2242 | 0.967755 | 0.9146 | 0.388084 | 0.0959 | 0.10221 | 3.1351 | 0.285478 | 1.1444 | 0.043534 | 10.5568 |
| G08 | hsa-miR-613 | 0.006874 | 0.2304 | 0.437628 | 0.8391 | 0.264278 | 0.3769 | 0.502481 | 0.4989 | 0.442758 | 0.3496 | 0.298887 | 0.732 |
| G09 | hsa-miR-7 | 0.355029 | 0.5569 | 0.077829 | 6.5304 | 0.324229 | 1.0485 | 0.309827 | 36.4571 | 0.312311 | 41.4747 | 0.162463 | 133.7477 |
| G10 | hsa-miR-93 | 0.316455 | 0.4698 | 0.777679 | 0.4518 | 0.253746 | 1.111 | 0.325764 | 0.0434 | 0.056959 | 0.0347 | 0.526678 | 0.0226 |
| G11 | hsa-miR-96 | 0.443686 | 0.3356 | 0.712408 | 0.9139 | 0.201963 | 0.2023 | 0.073528 | 9.5262 | 0.145595 | 5.8007 | 0.08046 | 19.2974 |
| G12 | hsa-miR-98 | 0.702742 | 0.5844 | 0.067456 | 0.3121 | 0.042553 | 0.3994 | 0.49985 | 0.4132 | 0.581791 | 0.2798 | 0.457251 | 0.3102 |
| H01 | cel-miR-39 | 0.488732 | 0.5845 | 0.33601 | 0.1482 | 0.262164 | 0.5936 | 0.370556 | 0.0028 | 0.203107 | 0.0015 | 0.200132 | 0.001 |
| H02 | cel-miR-39 | 0.408702 | 1.5477 | 0.521749 | 0.6441 | 0.334744 | 1.2376 | 0.51696 | 0.7004 | 0.794116 | 0.7855 | 0.166034 | 0.4452 |
| H03 | SNORD61 | 0.803037 | 1.0886 | 0.163031 | 0.5865 | 0.680534 | 0.9993 | 0.107427 | 0.5094 | 0.292989 | 0.5498 | 0.016908 | 0.3409 |
| H04 | SNORD68 | 0.06804 | 2.2196 | 0.164338 | 0.6291 | 0.331418 | 1.0922 | 0.716634 | 0.7483 | 0.989905 | 0.7738 | 0.092594 | 0.5087 |
| H05 | SNORD72 | 0.606773 | 0.8918 | 0.843742 | 1.0474 | 0.86144 | 1.0396 | 0.233322 | 0.6418 | 0.198328 | 0.6062 | 0.190527 | 0.6445 |
| H06 | SNORD95 | 0.97562 | 0.8899 | 0.50374 | 4.2857 | 0.934487 | 0.919 | 0.118052 | 105.5162 | 0.126895 | 141.2458 | 0.127929 | 287.7905 |
| H07 | SNORD96A | 0.58128 | 0.6632 | 0.57541 | 0.7403 | 0.345883 | 0.9151 | 0.139658 | 0.2418 | 0.029043 | 0.2042 | 0.213832 | 0.2064 |
| H08 | RNU6-2 | 0.325273 | 0.6567 | 0.630915 | 1.3436 | 0.707738 | 0.9873 | 0.109722 | 0.5992 | 0.022726 | 0.5308 | 0.353448 | 0.8156 |
| H09 | miRTC | 0.264305 | 0.4744 | 0.329091 | 6.6142 | 0.93581 | 0.7955 | 0.144349 | 208.8246 | 0.178838 | 288.4283 | 0.197104 | 937.2365 |
| H10 | miRTC | 0.457798 | 0.5673 | 0.122856 | 0.1055 | 0.071324 | 0.4741 | 0.990633 | 0.003 | 0.588466 | 0.0015 | 0.21516 | 0.0009 |
| H11 | PPC | 0.334376 | 1.2217 | 0.151228 | 0.6724 | 0.96426 | 1.1289 | 0.298073 | 2.7822 | 0.34295 | 2.9743 | 0.046452 | 2.1053 |
| H12 | PPC | 0.159271 | 1.4963 | 0.01215 | 0.4427 | 0.781375 | 0.7492 | 0.261077 | 1.5654 | 0.274705 | 1.6461 | 0.765472 | 0.9039 |
| A01 | hsa-let-7a | 0.469253 | 1.2454 | 0.451748 | 1.1716 | 0.465739 | 1.1461 | 0.332273 | 1.2693 | 0.271989 | 1.398 | 0.342044 | 1.2768 |

p values less than 0.05 are indicated in red. P-value ≤ 0.05 was considered as statistically significant

Fold-Change (2^(- Delta Delta Ct)) is the normalized gene expression (2^(- Delta Ct)) in the Test Sample divided the normalized gene expression (2^(- Delta Ct)) in the Control sample.

Fold-Change values greater than two indicate an up-regulation.

Fold-change values less than 0.5 indicate a down-regulation.
