# Supplementary material for: Identification of miR-10b, miR-26a, miR-146a and miR-153 as potential triple-negative breast cancer biomarkers
Source: Cell Oncol (Dordr). 2015 Sep 21;38(6):433–42. doi: 10.1007/s13402-015-0239-3 (PMC4653246; doi:10.1007/s13402-015-0239-3)
Supplement: Supplementary file 12 — (DOCX 13 kb) [file 13402_2015_239_MOESM8_ESM.docx]

| **Algorithmes** | ***Miranda (2008)*** | ***Microcosm Targets (2008)*** | ***Pictar (2005)*** | ***Tarbase (2009)*** | ***TargescanHuman 6,1 (2012)*** | |
| --- | --- | --- | --- | --- | --- | --- |
| **miARNS** |  |  |  |  | **Mammaires** | **Vertebrés** |
| hsa-miR- |  |  | 30a-3p |  | 105 | 1420ac |
| hsa-miR- | 1 | 17 | 132 | 16 | 122/122a | 146ac/146-5p |
| hsa-miR- | 7 | 20 | 140 | 21 | 125a-3p | 17 / 17-5p |
| hsa-miR- | 125a-3p | 28 | 143 | 24 | 129-5p/129ab-5p | 199ab-5p |
| hsa-miR- | 132 | 32 | 154 | 192 | 132 | 205/205ab |
| hsa-miR- | 141 | 92 | 185 | 212 | 150 | 20ab/20b-5p |
| hsa-miR- | 153 | 99* | 197 | 335 | 153 | 212/212-3p |
| hsa-miR- | 186 | 122 | 205 | 146a | 186 | 216b/216b-5p |
| hsa-miR- | 190 | 146 | 370 | 15a | 197 | 218/218a |
| hsa-miR- | 206 | 153 |  | 193b | 224 | 24/24ab/24-3p |
| hsa-miR- | 212 | 187 |  |  | 294 | 26ab |
| hsa-miR- | 218 | 200 |  |  | 295 | 28-5p |
| hsa-miR- | 224 | 220 |  |  | 302 | 291a-3p |
| hsa-miR- | 543 | 224 |  |  | 313 | 448-3p |
| hsa-miR- | 613 | 333 |  |  | 372 | 518a-3p |
| hsa-miR- | 146a | 367 |  |  | 373 | 519a |
| hsa-miR- | 146b-5p | 373 |  |  | 411 | 519d |
| hsa-miR- | 190b | 378 |  |  | 427 | 520acd-3p |
| hsa-miR- | 200a | 464 |  |  | 428 | 520bc |
| hsa-miR- | 216b | 516 |  |  | 448 | 590-3p |
| hsa-miR- |  | 518 |  |  | 708 | 7/7ab |
| hsa-miR- |  | 526 |  |  | 758 | 9/9ab |
| hsa-miR- |  | 540 |  |  | 1297 | 93/93a |
| hsa-miR- |  | 701 |  |  | 1378 |  |
| hsa-miR- |  | 760 |  |  | 1407 |  |
| hsa-miR- |  | 125* |  |  | 1653 |  |
| hsa-miR- |  | 143* |  |  | 4465 |  |
| hsa-miR- |  | 20* |  |  | 5127 |  |
| hsa-miR- |  |  |  |  | 106a |  |
| hsa-miR- |  |  |  |  | 106ab |  |

**Table S4** microRNAs predicted to bind the 3’UTR of the BRCA1 gene by five algorithms
